# Targeting TGF-β–Smad2/3–JNK1-mediated SIRT1 activity overcomes the chemoresistance of KRAS mutation lung cancer

**DOI:** 10.1038/s12276-025-01536-8

**Published:** 2025-09-12

**Authors:** Dong Hoon Shin, Minyoung Choi, Chungyong Han, Sang Soo Kim

**Affiliations:** 1 Research Institute, National Cancer Center, Goyang-si, Republic of Korea; 2 Cancer Biomedical Science, Graduate School of Cancer Science and Policy, National Cancer Center, Goyang-si, Gyeonggi-do Republic of Korea

**Keywords:** Non-small-cell lung cancer, Extracellular signalling molecules

## Abstract

Patients with lung cancer harboring a KRAS oncogenic driver mutation have a very poor prognosis. Recently, we reported that SIRT1 is upregulated by the KRAS^Mut^–c-Myc axis, and that KRAS^Mut^-induced SIRT1 is stably deacetylated at lysine 104, which in turn increases KRAS^Mut^ activity and enhances chemoresistance. Notably, SIRT1 activity as well as SIRT1 levels are more elevated in KRAS^Mut^ cells compared with EGFR^Mut^, KRAS^Mut^- and EGFR^Mut^-negative cells, and nontumorigenic cells. This prompted us to investigate the mechanism by which SIRT1 activity was increased and the role of pSIRT1 in the chemoresistance of KRAS^Mut^ lung cancer cells. The activated MEK–ERK pathway under KRAS^Mut^ increased AP-1 transcription activity, which in turn enhanced TGF-β1 secretion. The secreted TGF-β1 activated the Smad2/3–JNK1 signaling pathway in an autocrine manner, increasing pSIRT1^S27^ and pSIRT1^S47^, ultimately enhancing KRAS^Mut^ activity through KRAS deacetylation and affecting chemoresistance. We identified a small molecule from the natural compound library—Kuwanon C (KWN-C), a SIRT1 activity inhibitor—which reduced pSIRT1^S27^ and pSIRT1^S47^ levels via a decrease in the activity of the TGF-β1–-Smad2/3–JNK1 signaling pathway. Treatment with the SIRT1 activity inhibitor triggered the anticancer effects of cisplatin and pemetrexed in human lung cancer cells, lung orthotopic tumors and a spontaneous in vivo model of KRAS^Mut^ lung cancer. Our findings reveal a novel pathway critical for the regulation of SIRT1 activity in KRAS^Mut^ lung cancer and provide important evidence for the potential application of SIRT1 activity inhibitors as an adjuvant chemotherapy, overcoming chemoresistance in patients with KRAS^Mut^ lung cancer.

## SIRT1 inhibition enhances chemotherapy in KRAS lung cancer

Mutations in the KRAS gene are common in several cancers, including lung cancer, and make treatment challenging. Researchers explore a new approach by focusing on a protein called SIRT1, which is found at higher levels in KRAS-mutated lung cancer cells. They used various laboratory techniques to study how SIRT1 interacts with KRAS mutations. They discovered that SIRT1 helps KRAS mutations become more active, which contributes to cancer growth and resistance to chemotherapy. By using natural compounds to inhibit SIRT1 activity, they found that they could make cancer cells more sensitive to existing chemotherapy drugs. The study suggests that targeting SIRT1 could be a promising strategy to improve treatment for patients with KRAS-mutated lung cancer. They propose that combining SIRT1 inhibitors with current chemotherapy could enhance treatment effectiveness and reduce drug resistance.

This summary was initially drafted using artificial intelligence, then revised and fact-checked by the author.

## Introduction

Mutations in Kirsten rat sarcoma viral oncogene homolog (KRAS) are among the most common genomic alterations identified in solid tumors, especially in pancreatic ductal adenocarcinoma, colorectal cancer and lung cancer^1^. Lung cancer is the most prevalent cause of cancer-related death worldwide, while non-small cell lung cancer (NSCLC) is the most frequent lung cancer subtype. One of the most frequent driver mutations for NSCLCs involves the activation of mutations in KRAS, approximately 8–24% (refs. ^2,3^). The KRAS protein is a small GTPase with a binary molecular switch function that activates rapidly accelerated fibrosarcoma kinase (RAF), mitogen-activated protein kinase (MEK) and phosphatidylinositol 3-kinase (PI3K) signaling of downstream pathways. These signaling cascades induced by mutant KRAS enhance the proliferation and differentiation of NSCLC cells^4^. For example, spontaneous lung adenocarcinoma is triggered by the conditional expression of the oncogenic Kras^G12D^ allele in the mouse airway epithelium^5^. Consequently, current research has been focusing on rendering an ideal targeting KRAS mutations therapeutically. However, it is very challenging to develop small-molecule inhibitors, because mutant KRAS has a very high affinity for guanosine triphosphate (GTP) and the catalytic sites are very small and difficult to target. Initial therapeutic approaches attempting to indirectly target the posttranslational modification of KRAS, such as inhibiting KRAS membrane localization with farnesyltransferase inhibitors (FTIs), failed in clinical trials and are no longer considered successful^6^. In addition, downstream pathways of KRAS—such as RAF, MEK and PI3K—have also been targeted for therapeutic blockade^7^. However, these efforts have not been successful in clinical trials over the past 30 years. Recently, Amgen and Mirati therapeutics have developed two direct KRAS^G12C^ inhibitors that act by selectively forming a covalent bond with cysteine 12 within the switch II pocket of KRAS^G12C^ protein, thereby locking KRAS in the inactive state to arrest cell proliferation^8^. However, emerging preclinical and clinical evidence suggests that an additional obstacle to KRAS^G12C^ inhibitor treatment is the inevitable emergence of drug resistance^9^. Consequently, there is a pressing need to identify druggable cooperating partners for oncogenic KRAS mutation therapy^10^.

Previously, we identified a druggable therapeutic partner, Sirtuin 1 (SIRT1), for combination treatment with existing chemotherapy for a patient with KRAS^Mut^ cancer. Sirtuins belong to the class III histone deacetylase (HDAC) family that also includes SIRT1–7 isoforms in mammalian cells^11^. Among these, SIRT1 requires NAD^+^ as a substrate to deacetylate the lysine residues in both histones and nonhistone proteins, such as p53^12^. Interestingly, SIRT1 plays a dual role in tumor progression, both as a tumor suppressor and promoter, and its role is independent of the actual SIRT1 levels^13^. For example, previous studies found that SIRT1 expression is decreased in human breast, liver and colon tumors^14,15^. Conversely, increased SIRT1 expression has been correlated with favorable outcomes in human head and neck squamous cell carcinomas and ovarian tumors^16,17^. Furthermore, reduced SIRT1 expression has been found to accelerate tumor development in a p53-heterozygous background^14^, whereas its overexpression could block the growth of different types of tumors in cell culture experiments^18^ and mouse models^19^. SIRT1 deacetylation activity can regulate gene transcription and influences diverse, yet critical, biological processes, such as cell division, differentiation, senescence and tumorigenesis^20^. Intriguingly, we identified that SIRT1 activity was increased specifically in KRAS^Mut^ lung cancer cells compared with EGFR^Mut^ lung cancer or nontumorigenic cells. Moreover, we discovered the mechanism that enhanced SIRT1 activity in KRAS^Mut^ lung cancer and screened natural product-derived SIRT1 activity inhibitors that could subsequently be combined with existing chemotherapy regimens. Notably, naturally occurring compounds play an essential role in the treatment of various types of cancer because they can maximize the anticancer effects of first-line chemotherapy, while simultaneously minimizing potential side effects^21^.

Previously, we biochemically investigated the functional interactions between SIRT1 and KRAS^Mut^, and we found that SIRT1 is specifically deacetylated at lysine 104 in KRAS^Mut^ lung cancer cells. Besides, SIRT1 expression is dependent on KRAS^Mut^ activation and, in turn, increases KRAS mutation activity and chemoresistance in KRAS^Mut^ lung cancer. In theory, a decrease in SIRT1 activity may sensitize KRAS^Mut^ lung cancer cells to anticancer drugs. Therefore, we screened natural product-derived SIRT1 activity inhibitors that may maximize the efficacy of antineoplastic agents while minimizing side effects. In particular, we focused on why SIRT1 activity increases in KRAS^Mut^ lung cancer and evaluated the role of a SIRT1 activity inhibitor derived from natural products in the chemoresistance of KRASMut lung cancer.

## Materials and methods

### Cell lines and culture conditions

The H358, H460, A427, NCIH727, NCIH23, SKLU-1, SW960 (KRAS^Mut^), H1650 (KRAS^Mut^ and EGFR^Mut^), H1975, HCC827, HCC2279, PC9 (EGFR^Mut^), HCC1666, H322M, H522, Calu-3 (KRAS^WT^ and EGFR^WT^), BEAS-2B (normal human bronchial epithelial cell) and human embryonic kidney (HEK)-293T cell lines were obtained from the American Type Culture Collection. The cells were cultured in either HyClone RPMI-1640 or Dulbecco’s modified Eagle medium (Thermo Fisher Scientific), each containing 10% fetal bovine serum (FBS) and 1% penicillin and streptomycin. Cells were maintained in a 37 °C humidified incubator with 5% CO_2_ in air. All cell lines were authenticated by short tandem repeat analysis and were free of mycoplasma contamination.

### Plasmids, antibodies, chemicals and ELISA assay kits

*KRAS*^*WT*^ (#75282), *KRAS*^*G12C*^ (#58901), *KRAS*^*G12D*^ (#58902), *KRAS*^*G12V*^ (#46746) and *JNK1-GFP* (#86830) plasmids were purchased from Addgene. Small interfering RNAs (siRNAs), including siKRAS (sc-35731 and sc-43874), siSIRT1 (sc-40986) and siSmad2/3 (sc-37238) and antibodies including SIRT1 (sc-15454), KRAS (F234, sc-30), PARP (sc-8007) and cleaved PARP (sc-56196) were purchased from Santa Cruz Biotechnology. Antibodies against MKK4 (#9152), pMKK4 (Ser257/Thr261) (#9156), MKK7 (#4172), pMKK7 (Ser271/Thr275) (#4172), JNK1 (#3708), pJNK1 (Thr183/Tyr185) (#4668), pSIRT1 (Ser27) (#2327), pSIRT1 (Ser47) (#2314), Smad2/3 (#3102), pSmad2 (Ser465/467)/Smad3 (Ser423/425) (#8828), ERK1/2 (#4695), pERK (T202/Y204) (#4370), pAkt (S473) (#4060), Akt (#4685), cleaved caspase-3 (Asp175) (#9661) and β-actin (#3700) were obtained from Cell Signaling Technology. Anti-acetylation lysin (ab190479) and terminal deoxynucleotidyl transferase dUTP nick-end labeling (TUNEL) assay kit-HRP-DAB (ab206386) were obtained from Abcam. pSIRT1 (Thr530) (SR1099) was purchased from Novus. Human TGFβ-1 enzyme-linked immunosorbent assay (ELISA) kit (BMS249-4) and pJNK1 (Thr183, #PA5-37698) antibody were purchased from Thermo Fisher Scientific. The KRAS activation assay kit (STA-400-K) was obtained from Cell Biolabs. Cisplatin (CP; S1166), pemetrexed (MTA; S5971), anisomycin (S7409) and SP600125 (S1460) were purchased from Selleck Chemicals. Natural product compounds libraries LBP0004–LBP0013 were provided by the National Institute for Korean Medicine Development (NIKOM).

### Transfection

For transient overexpression or knockdown, H358, H460, A427, H727, NCIH23, SKLU-1 and HEK-293T cells growing at 40% density were transfected with plasmids (2 μg per 60-mm dish) or siRNAs (80 nM) using Lipofectamine 2000 from Thermo Fisher Scientific. The transfected cells were allowed to stabilize for 48 h before use in experiments.

### In vitro kinase assay

Human recombinant protein SIRT1 (CUSABIO, CSB-EP822202HU) (10 μM) were incubated in 1× kinase buffer (Cell Signaling), supplemented with 2 mM ATP (Cell Signaling), and 25 ng/μl purified active human JNK1 (Abcam, ab201870). Reactions (20 μl total volume) were incubated at 32 °C for 30 min. The reaction was stopped by the addition of 5 μl 6× sodium dodecyl sulfate–polyacrylamide gel electrophoresis (SDS–PAGE) sample buffer and boiled at 95 °C for 5 min. Samples were analyzed by immunoblotting assay.

### AP-1 luciferase assay

AP-1 promoter luciferase vector was from Addgene (#11783), and cells were transfected with AP-1 promoter with a firefly luciferase promoter as a reference by Lipofectamine 2000 reagent from Thermo Fisher Scientific (cat. no. 11668019). Then, 24 h after transfection, total cell lysates were collected to measure the dual luciferase activity of Renilla and firefly luciferase. The relative luciferase activity value was calculated by normalizing the activity of firefly luciferase to Renilla luciferase.

### KRAS activation assay

The active GTP-bound KRAS was quantified using a KRAS activation assay kit (cat. no. STA-400-K, Cell Bio Labs) according to the manufacturer’s instructions. In brief, 1 ml of total cell lysate (2.0 mg) was incubated with 40 μl of Raf-1–RBD agarose beads for 2 h at 4 °C to pull down the activated GTP-bound KRAS. The bead pellet was washed three times with 0.5 ml of 1× assay buffer, centrifuged and aspirated each time. After the last wash, bead pellet was resuspended with 40 μl of 2× reducing SDS–PAGE sample buffer. Samples were electrophoresed, transferred to a polyvinylidene fluoride membrane and blotted using KRAS antibody (Abnova, cat. no. H00003845-M01) to determine the KRAS protein levels.

### SIRT1 activity assay

SIRT1 stably expressed HEK-293T cells were treated with natural product compounds for 3 days. These 1 × 10^6^ cells were added to 200 μl cold homogenization buffer containing 1,4-dithiothreitol (DTT) and protease inhibitor cocktail and homogenize cells on ice. The cell homogenate was transferred including cell debris to a cold microfuge tube and agitated on a rotary shaker at 4 °C for 15 min. The cell homogenate was centrifuged at 16,000*g* for 20 min at 4 °C. The clarified supernatant was transferred to a fresh prechilled tube and kept on ice. To make 7-amino-4-trifluoromethylcoumarin (AFC) standard, AFC standard was diluted to 10 μM by adding 10 μl of 1 mM AFC standard to 990 μl of SIRT1 assay buffer with DTT. Then, 0, 20, 40, 60, 80 and 100 μl of diluted 10 μM AFC standard was added into individual wells in a 96-well white plate, and the volume was adjusted to 100 μl per well with SIRT1 assay buffer with DTT to generate 0, 200, 400, 600, 800 and 1,000 pmol per well of AFC standard, respectively. Fifty microliters of cell lysates was added into desired wells in a 96-well plate. For the positive control, 2 μl of positive control was added in the desired well. The volume of samples and positive control should be made up to 50 μl per well with SIRT1 assay buffer containing DTT. Reaction mixture (SIRT1 assay buffer with DTT 36 μl, substrate 2 μl and NAD 2 μl) was added into each sample, positive control and background control well. After mixing well, the plates were incubated at 37 °C for 60 min; then 10 μl of developer was added to each well except standards, and the contents were mixed, followed by incubation for 15 min at 37 °C. Fluorescence was measured (Ex/Em = 400/505 nm) in end point mode. SIRT1 activity calculation was represented (Supplementary Fig. 1).

### Cell viability and colony-forming assays

For the viability assay, 2 × 10^3^ cells were plated in 96-well plates and incubated with medium containing the drugs for 3 days. Cell viability was measured using CellTiter 96 AQueous One Solution Reagent (Promega, cat. no. G9241) containing a tetrazolium compound (3-(4,5-dimethylthiazol-2-yl)-5-(3-carboxymethoxyphenyl)-2-(4-sulfophenyl)-2*H*-tetrazolium, inner salt; MTS) at 490 nm for 90 min (VERSAmax). Six replicate wells were used for each analysis, and at least three independent experiments were performed. To analyze anchorage-independent growth, cells (2 × 10^3^ cells per well) were suspended in 0.4% top agar and cultured on 0.8% agar for 40 days. The cells were stained with crystal violet, and cell masses (0.2 mm diameter) were counted as colonies.

### Immunoblotting and immunoprecipitation

Proteins obtained from cell extracts were separated by SDS–PAGE and then transferred to Immobilon-P membranes (Millipore, cat. no. IPVH85R). The membranes were blocked with 5% nonfat milk, incubated overnight at 4 °C with primary antibodies diluted 1:100–1,000 and then incubated for 1 h at room temperature with the corresponding horseradish peroxidase-conjugated secondary antibodies. Antigen–antibody complexes were visualized using SuperSignal West Femto luminol enhancer solution (Thermo Fisher Scientific, cat. no. 34094). For immunoprecipitation, cell lysates were incubated with 5 μl of antibody, or preimmune serum, at 4 °C for 2 h. Immune complexes were further incubated with protein A/G-Sepharose beads (Thermo Fisher Scientific, cat. no. IP10) at 4 °C for 4 h. The immunocomplexes were eluted by boiling for 10 min in sample buffer containing 2% SDS and 10 mM DTT, subjected to SDS–PAGE and then immunoblotted using anti-rabbit and anti-mouse antibodies.

### Quantitative real-time PCR

Total cellular RNA was extracted with TRIzol (Thermo Fisher Scientific, cat. no. T9424), using chloroform, precipitated with isopropyl alcohol, washed with 70% ethanol and eluted in RNase-free water. The concentration of the isolated RNA was measured using a Nanodrop 2000 spectrophotometer (Thermo Fisher Scientific, cat. no. ND2000CLAPTOP) at 260 nm. The cDNAs used as template in quantitative real-time PCR (RT–qPCR) were prepared using 1,000 ng of total-RNA. The mRNA expression was evaluated by RT–qPCR using Sensi FAST SYBR (Bioline, cat. no. BIO-98005) and normalized to RPL32 in each sample. For PCR, DNA polymerase activation was performed for 5 min at 94 °C, and amplification was then conducted in a Light Cycler 96 instrument (Roche Diagnostics). The primer sequences used in these experiments are listed in Table 1.

**Table 1:** Primer sequences for RT–qPCR.

Gene	Forward primer	Reverse primer
*SIRT1*	TAGCCTTGTCAGATAAGGAAGGA	TGTTCTGGGTATAGTTGCGAAGT
*Sirt1*	GGAGCAGATTAGTAAGCGGCTTG	GTTACTGCCACAGGAACTAGAGG
*TGF-β1*	TACCTGAACCCGTGTTGCTCTC	GTTGCTGAGGTATCGCCAGGAA

All reactions were carried out in triplicate. A comparative threshold cycle (ΔCT) method was used to compare values expressed as 2^−ΔΔCT^.

ΔCT = CT (target gene) – CT (reference gene) ΔΔCT = ΔCT (target sample) − ΔCT (reference sample).

### Immunohistochemical staining

Paraffin-embedded sections (5 μm thick) were deparaffinized, and heat-induced epitope retrieval was performed using targeted retrieval solution 9 (Dako, cat. no. RE7119). The slides were treated with 3% hydrogen peroxide for 20 min to block endogenous peroxidase activity, followed by washes with deionized water for 3 min. The slides were then incubated with 0.5% bovine serum albumin blocking solution for 1 h at room temperature and then incubated with primary antibodies against pJNK1, cleaved caspase-3, cleaved RPAR and TUNEL mixture overnight at 4 °C. Immunoreactions were detected using the VECTASTAIN ABC HRP Kit (Burlingame, cat. no. PK-4000). Hematoxylin staining was used for counterstaining.

### TUNEL assay

H358 and H460 cells were treated with different concentrations of drugs collected and processed for flow cytometry (FACS Fortessa, Becton Dickinson) using the APO-BrdUTM TUNEL Assay Kit with Alexa Fluor 568 anti-BrdU (Thermo Fisher Scientific, cat. no. A23210), according to the manufacturer’s instructions. TUNEL is a method for detecting DNA fragments during the cell cycle by labeling the 3’-hydroxyl termini of DNA double-strand breaks. Floating and attached cells were fixed with 4% paraformaldehyde and subsequently incubated with a DNA-labeling solution containing BrdU. After washing, the samples were incubated with Alexa Fluor-labeled anti-BrdU, pelleted and washed with the wash buffer. Cells were incubated with RNase/propidium iodide (PI) solution (provided in the kit) and incubated at 37 °C for 15 min. The cells were analyzed by flow cytometry, and the data were expressed as the percentage of apoptotic cells (in the total cells). Each experiment was performed in triplicate.

### PI-Annexin V apoptosis assay

Apoptosis was also evaluated using annexin V&PI staining with flow cytometry. After treatment with different combinations of drugs, cells were collected and centrifuged at 300*g* for 5 min and then washed twice with cold phosphate-buffered saline (PBS). Cells were then resuspended in binding buffer and incubated with PI and annexin V-FITC (BD Biosciences, cat. no. RUO-556547) for 15 min at 21 °C. A minimum of 10,000 events were collected and analyzed by flow cytometry using a BD LSR Fortessa analyzer (BD Biosciences).

### Lung orthotopic mouse model

All animal procedures were performed in accordance with a protocol approved by the Institutional Animal Care and Use Committee of the National Cancer Centre Research Institute. The National Cancer Centre Research Institute is an Association for Assessment and Accreditation of Laboratory Animal Care International (AAALAC International)-accredited facility and abides by the Institute of Laboratory Animal Resources guide and Usage Committee. Nude mice (BALB/cAnNCrj-nu/nu) from Charles River Laboratories Japan were anesthetized with isoflurane via inhalation in an enclosed boxed chamber. Mice were positioned in a supine position, and the jaw and tongue were drawn away from the esophageal region using forceps while inserting a 22-gauge Hamilton TLC syringe (Thermo Fisher Scientific, cat. no. 1705) into the trachea. Light was shown on the mouse’s upper chest, and then the mice were injected with 1 × 10^6^ cancer cells suspended in 100 μl of PBS. After injection, the mice were allowed to recover before being replaced back in the cage for a predetermined period after exposure. Lung tumor bioluminescence imaging was performed after injection to quantify lung tumor burden using IVIS Lumina XRMS In Vivo Imaging System (PerkinElmer).

### Genetically engineered LSL-Kras^G12D/+^ mouse model

Lox-stop-lox (LSL)-Kras^G12D/+^ (B6.129S4-*Kras*^*tm4Tyj*^/J, stock #008179) mice on a C57BL/6J background were purchased from The Jackson Laboratory. Initially, LSL-Kras^G12D/+^ mice were anesthetized with isoflurane via inhalation in an enclosed boxed chamber and infected with 5 × 10^7^ infectious particles of Ad-Cre (HanBio) per mouse via intranasal injection. After cre-adenovirus infection, KRAS^G12D^ mice were treated with CP, MTA and/or kuwanon C (KWN-C) intraperitoneally (i.p.). Mice were euthanized with CO_2_ asphyxiation. After lung perfusion with PBS, the mouse lungs were collected from dimethyl sulfoxide (DMSO)- and drug-treated groups and immediately fixed in 10% neutral buffered formalin (Thermo Fisher Scientific) and embedded in paraffin blocks. Lung tissues representing different regions of the lung were vertically put into paraffin blocks for hematoxylin–eosin (H&E) staining. Lung tissue samples were sectioned at 4 μm three times for placement of slides and stained with H&E. H&E staining samples were scanned using Nano Zoomer 2.0-HT, and images were analyzed using ImageScope viewing software (Leica Biosystems). Tumor numbers were counted under a microscope, and tumor area was quantified using Openlab modular imaging software (PerkinElmer).

### Mouse blood analysis

Whole blood (50 μl) was collected in ethylenediaminetetraacetic-acid-coated tubes via mouse orbital sinus injection of anesthetized mice for hematology studies. Specimens were analyzed for white blood cell (WBC), red blood cells (RBC) and platelets (PLT) by BC-5000 Vet auto hematology analyzer (Shenzhen Mindary Animal Medical Technology). Alanine aminotransferase (ALT) (MAK464), aspartate transaminase (AST) (MAK467) and blood urea nitrogen (BUN) (EIABUN) were analyzed using assay kits from Thermo Fisher Scientific.

### Statistical analysis

Results are expressed as the mean ± standard error of the mean (s.e.m.) or standard deviation (s.d.) from more than three independent samples, as calculated using Microsoft Office LTSC Professional Plus Excel 2021 and SPSS statistical software package. Groups were compared using two-tailed, unpaired Student’s *t*-tests for all assays, and statistical tests were two-sided. The nonparametric statistical tests used in each case were the Mann–Whitney or chi-square tests. Statistical significance was set at *P* < 0.05.

## Results

### *KRAS*^*Mut*^ increases SIRT1 expression in *KRAS*^*Mut*^ NSCLCs and *Kras*^*G12D*^ Tg mouse lung tumor

**Fig. 1:**
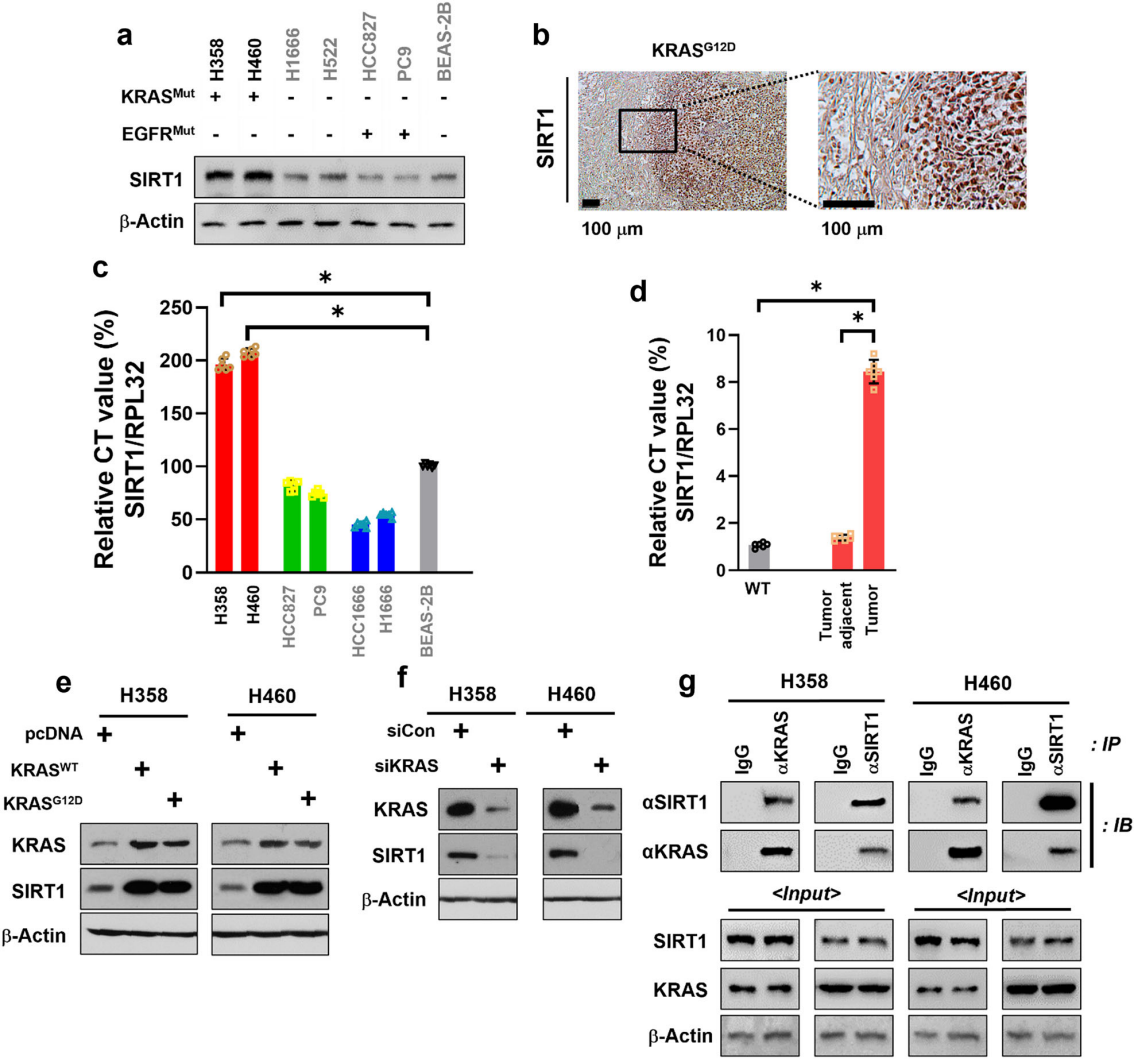
KRAS^Mut^ regulates SIRT1 expression in NSCLC cells both in vitro and in vivo. **a** Normal human bronchial epithelial cell (BEAS-2B), KRAS^Mut^ cell lines (H358 and H460), KRAS^WT^ and EGFR^WT^ cell lines (HCC1666 and H522), and EGFR^Mut^ cell lines (HCC827 and PC9) were collected with lysis buffer, and immunoblotted with anti-SIRT1 and β-actin antibody. **b** Immunohistochemical staining for SIRT1 with the lung from LSL-Kras^G12D^ Tg mouse at 16 weeks after administration of adenovirus Cre recombinase induction. Representative images are shown. Scale bar, 100 μm. High-magnification images correspond to the areas marked by the black box. **c** SIRT1 mRNA expression was measured by RT–qPCR with the same cell lines as in **a**. *RPL32* was used as internal control and for normalization. Student’s *t*-test, mean ± s.d.; *n* = 6, *P < 0.05. **d** The mRNA expression of Sirt1 was analyzed by RT–qPCR with the cancerous and adjacent noncancerous lung tissues of LSL-*Kras*^*G12D*^ Tg mouse. *Rpl32* was used as internal control and for normalization. Student’s *t*-test, mean ± s.e.m.; *n* = 6, *, *P* < 0.05. **e** H358 and H460 cells were transfected with the 2-μg plasmids of *pcDNA*, *KRAS*^*WT*^ and *KRAS*^*G12D*^. The cells were collected with cell lysis buffer and subjected to western blotting with anti-KRAS, anti-SIRT1 and β-actin antibodies. **f** H358 and H460 cells were transfected with siCon and siKRAS (80 nM). The cells were collected with cell lysis buffer and subjected to western blotting with same antibodies in **e**. **g** H358 and H460 cell extracts were immunoprecipitated with anti-KRAS and anti-SIRT1, and immunoblotted with anti-KRAS, anti-SIRT1 and β-actin antibodies.

It was previously reported that high levels of SIRT1 expression enhance tumorigenesis and are associated with poor prognosis in patients with NSCLC^22^. Therefore, we measured the expression of SIRT1 in a panel of human NSCLC cells and nontumorigenic lung epithelial cells using western blotting (Fig. 1a) and RT–qPCR (Fig. 1c). Notably, both protein and mRNA levels of SIRT1 were significantly higher in *KRAS*^*Mut*^ cell lines than in normal lung epithelial cells, as well as in *KRAS*^*Mut*^-negative and *EGFR*^*Mut*^-positive cell lines. To confirm the results of SIRT1 expression in NSCLC cell lines, we used LSL-KRAS^G12D^ mice as previously described^23^. By engineering LoxP DNA elements into the mouse genome that surround a synthetic ‘STOP’ element (LSL) inserted in front of KRAS^G12D^, Cre recombinase was delivered to activate KRAS^G12D^^24^. To control the expression of KRAS^G12D^, an LSL cassette was placed into the first intron of the *KRAS* gene. The LSL cassette consisted of transcriptional and translational stop elements flanked by LoxP sites that prevent the expression of the mutant allele until the stop elements are removed by the activity of Cre recombinase^25,26^. To produce KRAS^G12D^-driven lung cancer cells, alleles were induced in mouse lung using intratracheal injection of an adenoviral Cre recombinase. Primary lung tumors were developed at approximately 12 weeks post-inoculation. The protein and mRNA levels of Sirt1 were measured in the lung tumors of an LSL-*Kras*^*G12D*^ expression mouse model, a tumor-adjacent tissue and the lung tissue of *Kras*^*WT*^ at 16 weeks post-inoculation. The protein and mRNA expression of Sirt1 were substantially higher in *Kras*^*G12D*^ tumors than those in the adjacent tumors tissue and *Kras*^*WT*^ (Fig. 1b,d). To assess KRAS^Mut^-induced SIRT1 expression, we evaluated SIRT1 protein levels after the transfection of KRAS^WT^, KRAS^G12D^ plasmids and KRAS specific siRNA. The expressed *KRAS*^*WT*^ and *KRAS*^*G12D*^ plasmids were found to enhance SIRT1 protein levels in both H358 and H460 cells, independent of KRAS^WT^ and KRAS^Mut^ (Fig. 1e). By contrast, the downregulated KRAS expression by siKRAS was found to diminish SIRT1 protein levels (Fig. 1f). This result supports the notion that KRAS^Mut^ positively regulates SIRT1 expression, creating a KRAS^Mut^–SIRT1 feedback loop. Given that endogenous KRAS^Mut^ levels in H358 cells may be influenced by regulatory mechanisms, additional transfection of KRAS^Mut^ plasmids was necessary to evaluate whether enhanced KRAS expression directly affects SIRT1 levels. Furthermore, transfecting *KRAS*^*G12D*^ into H358^G12C^ and H460^Q61H^ cells allowed us to investigate whether different KRAS mutations exhibit consistent regulatory effects on SIRT1, reinforcing the hypothesis that SIRT1 activation is a general feature of KRAS-driven oncogenesis. Thus, this approach provided mechanistic validation while controlling for genetic background variations that exist among different KRAS^Mut^ cell lines. To further evaluate whether this regulation is mutation specific or simply due to increased total KRAS levels, we examined whether KRAS^WT^ and KRAS^G12D^ differentially regulate SIRT1 in a dose-dependent manner. In both H358 and H460 cells, KRAS^G12D^ induced significantly higher SIRT1 protein expression and enzymatic activity than KRAS^WT^ or pcDNA control at lower transfection doses (0.2–0.5 µg). However, at higher doses (1–2 µg), both KRAS^WT^ and KRAS^Mut^ yielded similar SIRT1 levels, probably due to transcriptional saturation (Supplementary Fig. 2a,b). These results suggest that the ability of KRAS^Mut^ to activate SIRT1 is more prominent under physiological, nonsaturating conditions and reflects a mutation-specific regulatory mechanism. To investigate whether KRAS^Mut^-induced SRIT1 affected KRAS^Mut^, co-immunoprecipitation with endogenous levels of KRAS^Mut^ and SIRT1 in H358 and H460 was performed. The interaction between KRAS^Mut^ and SIRT1 was crosschecked by changing the antibodies used for immunoprecipitation and immunoblotting (Fig. 1g).

To further validate whether the interaction between KRAS and SIRT1 is specific to KRAS^Mut^, we transfected equal amounts of KRAS^WT^ and KRAS^G12C^ plasmids into HEK 293 cells and performed co-immunoprecipitation assays. The results showed that SIRT1 binding was markedly stronger with KRAS^G12C^ compared with KRAS^WT^, even under equal plasmid DNA concentrations. Moreover, increasing the amount of transfected DNA led to a dose-dependent increase in the KRAS–SIRT1 interaction for both KRAS^WT^ and KRAS^G12C^; however, KRAS^G12C^ consistently showed a higher binding affinity for SIRT1 at all DNA levels tested (Supplementary Fig. 3). These findings indicate that, while both KRAS^WT^ and KRAS^Mut^ can interact with SIRT1, KRAS^Mut^ exhibits a stronger and potentially more stable interaction, supporting the presence of a mutation-specific feedback mechanism that enhances SIRT1 binding and downstream signaling activity.

It was previously reported that KRAS protein function is closely manipulated by posttranslational modification that directly regulates KRAS activity^27^. For example, KRAS acetylation at lysine 104 reduces guanine nucleotide exchange factor-mediated nucleotide exchange, making it difficult to reload GTP^28^. Given that KRAS^Mut^-induced SIRT1 binds to KRAS^Mut^ again, SIRT1 deacetylates KRAS acetylation at lysine 104, which increases KRAS^Mut^ activity. These results indicate that the mRNA and protein levels of SIRT1 were increased in NSCLC cells harboring KRAS^Mut^ and KRAS^Mut^-induced SIRT1 rebinds to KRAS^Mut^, thereby suggesting that KRAS^Mut^-induced SIRT1 makes a positive feedback loop with KRAS^Mut^ and influences KRAS^Mut^ activity regulation.

### Expressed SIRT1 by KRAS^Mut^ contributes to the chemoresistance of KRAS^Mut^ NSCLC

**Fig. 2:**
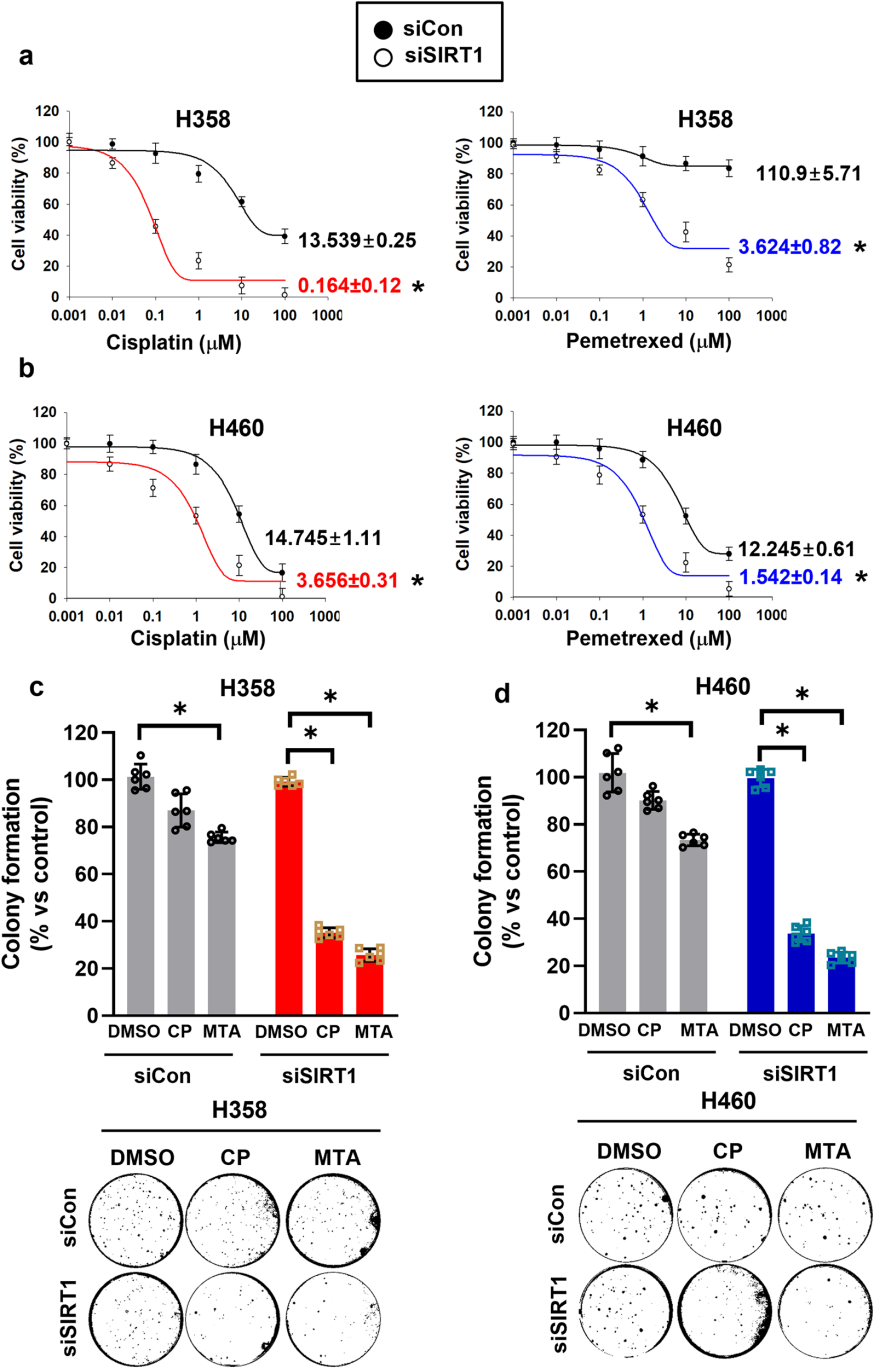
KRAS^Mut^-induced SIRT1 contributes to chemoresistance in KRAS^Mut^ NSCLC cells. **a**,**b** H358 and H460 cells were transfected with siCon and siSIRT1 and then treated with CP and MTA (0.001–100 μM). Cell proliferation was assessed using the MTS assay 3 days after the drug treatment. IC_50_ of CP and MTA was calculated on the basis of cell viability. Student’s *t*-test, mean ± s.d.; *n* = 6, *P < 0.05. **c**,**d** H358 and H460 cells were transfected and treated with CP (H358 1 μM and H460 5 μM) and MTA (H358 10 μM and H460 5 μM). The cells were then seeded with 0.5% top agar and cultured in a mixture of medium, CP and MTA. Cell colonies were stained with crystal violet and counted per 3.8 cm^2^. Student’s *t*-test, mean ± s.d.; *n* = 6, *P < 0.05.

It was previously reported that patients with high SIRT1 expression were significantly more likely to be resistant to chemotherapy than those with low SIRT1 expression^29^. To confirm whether expressed SIRT1 in KRAS^Mut^ NSCLC is associated with chemoresistance, KRAS^Mut^ cell lines, H358 and H460 cells were treated with two chemotherapy drugs commonly used in the clinic to treat NSCLC—namely, a platinum-based agent (CP) and an antifolate antineoplastic agent (MTA)—after reducing SIRT1 levels using SIRT1-specific siRNA. CP half maximal inhibitory concentration (IC_50_)values of H358 and H460 cells were significantly sensitized (more than 82.5-fold and 4.03-fold) compared with the siCon transfected groups, and the MTA IC_50_ values of these two cells were also treated less (30.6-fold and 7.94-fold, respectively) than the groups transfected with siCon (Fig. 2a,b). Furthermore, the formation of colonies of H358 and H460 cells transfected with siCon showed chemoresistance with CP and MTA compared with the DMSO-treated group. However, the colony numbers of H358 and H460 cells with depleted SIRT1 levels were significantly inhibited by CP and MTA compared with those in the DMSO- treated group (Fig. 2c,d). These results clearly indicate an association between KRAS^Mut^ NSCLC chemoresistance and SIRT1 expression.

### SIRT1 activity is specifically augmented in KRAS^Mut^ cells via the JNK1 pathway

**Fig. 3:**
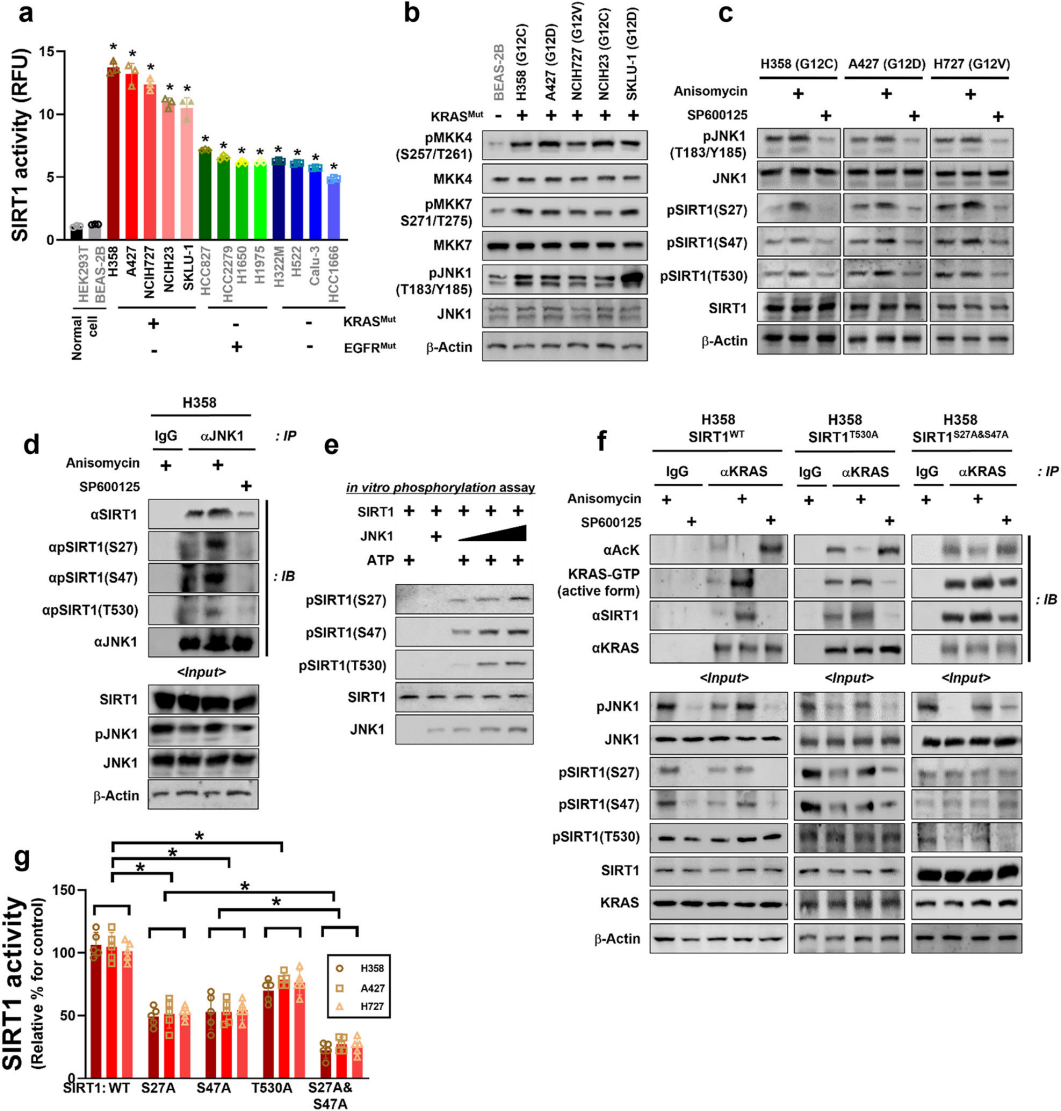
SIRT1 activity is increased by JNK1 in KRAS^Mut^ cells. **a** KRAS^Mut^ cell lines (H358, A427, NCIH727, NCIH23 and SKLU-1), EGFR^Mut^ cell lines (HCC827, HCC2279, H1650 and H1975), KRAS^WT^ and EGFR^WT^ cell lines (H322M, H522, Calu-3 and HCC1666) and nontumorigenic cells (HEK-293T and BEAS-2B) were collected with lysis buffer, and SIRT1 activity was measured with cell lysates. Student’s *t*-test, mean ± s.d.; *n* = 6, *P < 0.05. **b** Normal human bronchial epithelial cell (BEAS-2B) and KRAS^Mut^ cell lines used in **a** were collected with lysis buffer, and immunoblotted with anti-pMKK4, MKK, pMKK7, MKK, pJNK1, JNK1 and β-actin antibodies. **c** KRAS^Mut^ cell lines (H358, A427 and NCIH727) were treated with anisomycin 38 μM (10 μg/ml) (JNK1 activator) and SP600125 20 μM (JNK1 inhibitor) for 2 h. The protein levels of pJNK1, JNK, pSIRT1^S27^, pSIRT1^S47^, pSIRT1^T530^, SIRT1 and β-actin were measured by western blot analysis. **d** H358 cells were treated with anisomycin and SP600125 under the same condition as in **b** and then whole-cell lysates were subjected to immunoprecipitation with an anti-JNK1 antibody. Immunoblot analysis was performed using antibodies against SIRT1, pSIRT1^S27^, pSIRT1^S47^, pSIRT1^T530^, pJNK1, JNK and β-actin antibodies. **e** The recombinant proteins, SIRT1 and JNK1 were incubated in the reaction mixture for phosphorylation at 32 °C for 4 h, and then Ser- and Thr-phosphorylated peptides were identified using anti-pSIRT1^S27^, pSIRT1^S47^, pSIRT1^T530^ antibody. **f** H358 cells were transfected with *SIRT1*^*WT*^, *SIRT1*^*T530A*^, *SIRT1*^*S27A&S47A*^, and then H358 cell extracts were immunoprecipitated with anti-KRAS antibody and RAF-1 agarose beads and immunoblotted with anti-acetylation, anti-SIRT1, anti-KRAS, anti-KRAS–GTP-bound and β-actin antibodies. **g** KRAS^Mut^ cell lines (H358, A427 and H727) were transfected with *SIRT1*^*WT*^, *SIRT1*^*S27A*^, *SIRT1*^*S47A*^, *SIRT1*^*T530A*^ and *SIRT1*^*S27A,S47A*^ (2 μg), then collected with lysis buffer, and SIRT1 activity was assessed with cell lysates. Student’s *t*-test, mean ± s.d.; *n* = 6, *P < 0.05.

It was previously mentioned that KRAS^Mut^ was found to enhance SIRT1 expression. To determine whether KRAS^Mut^ also induces an increase in SIRT1 activity, we evaluated SIRT1 activity in a panel of human NSCLC and nontumorigenic cells. Notably, SIRT1 activity in KRAS^Mut^ cells showed the highest levels compared with EGFR^Mut^ and both negative cells of KRAS^Mut^ and EGFR^Mut^, even though SIRT1 activity in all NSCLC cells was increased more than that in nontumorigenic cells (Fig. 3a). One family member of mitogen-activated protein kinases under KRAS^Mut^ signaling, c-Jun N-terminal kinase 1 (JNK1), phosphorylates SIRT1 and promotes its enzymatic activity^30^. Therefore, we assessed the phosphorylation levels of MEK and JNK under KRAS downstream molecules. Our results revealed that the phosphorylation levels of JNK1 were increased in five KRAS^Mut^ cells compared with those in nontumorigenic lung epithelial cells through MKK4/7 phosphorylation (Fig. 3b). Next, the phosphorylation levels of three sites of SIRT1, namely S27, S47 and T530, were measured after treatment with an JNK1 activator or inhibitor to investigate whether SIRT1 activity was dependent on JNK1 phosphorylation. Our findings showed that JNK1 was activated in three KRAS^Mut^ types (G12C, G12D and G12V) in all three phosphorylation sites (Fig. 3c). To confirm these results, we prepared in vivo immunoprecipitation and in vitro phosphorylation assays, and our results showed that the JNK1 activator enhanced the interaction between JNK1 and SIRT1 and also increased the phosphorylation levels of S27 and S47 compared with those of T530. Conversely, the JNK1 inhibitor clearly diminished SIRT1 phosphorylation (Fig. 3d). In addition, the recombinant JNK1 peptide induced greater phosphorylation of the recombinant SIRT1 peptide at sites S27 and S47 compared with T530 (Fig. 3e). To examine which phosphorylation site had a greater effect on the acetylation and activation of KRAS^Mut^, we measured the H358 cells harboring three phosphorylation sites mutated to arginine. In *SIRT1*^*T530A*^ plasmid-transfected cells, the JNK1 activator reduced KRAS^Mut^ acetylation and increased KRAS^Mut^ activity owing to an increase in SIRT1 activity compared with the control, while JNK1 inhibitor showed a reverse effect of JNK1 activator compared with *SIRT1*^*WT*^ harboring H358 cells. In *SIRT1*^*S27A*^ and *SIRT1*^*S47A*^ plasmid-transfected cells, fluctuations were lower than in *SIRT1*^*T530A*^ plasmid-transfected cells, even though the acetylation and activity KRAS^Mut^ were either increased or decreased depending on the JNK1 activator or inhibitor, respectively (Fig. 3f). In accordance with our previous results, SIRT1 activity in *SIRT1*^*T530A*^ transfected cells showed the lowest decrease, approximately 20.6% versus the control, indicating that the effect induced by SIRT1^T530A^ on SIRT1 activity was lower than that by *SIRT1*^*S27A*^ and *SIRT1*^*S47A*^. In addition, SIRT1 activity in each transfection of *SIRT1*^*S27A*^ and *SIRT1*^*S47A*^ decreased by 47.8% versus the control. Intriguingly, both mutated *SIRT1*^*S27A*^ and *SIRT1*^*S47A*^ showed the lowest SIRT1 activity, that is, approximately 20.6% versus the control, indicating that SIRT1 activity phosphorylation by JNK1 was more dependent on *SIRT1*^*S27A*^ and *SIRT1*^*S47A*^ than on *SIRT1*^*T530A*^ (Fig. 3g). These results highlight that the activation of KRAS^Mut^–JNK1 pathway increases SIRT1 phosphorylation, especially at S27 and S47, thereby enhancing SIRT1 activity in KRAS^Mut^ cells and KRAS activity through an increase in KRAS deacetylation.

### SRIT1 activity inhibitor screening with natural product compounds

Drugs such as lovastatin, paclitaxel, silibinin and penicillin were derived either directly or indirectly from natural product compounds^31,32^. The subsequent statistical analyses revealed that all natural products had great potential as novel lead structures for drug discovery and development. Therefore, we screened 800 natural product-derived SIRT1 activity inhibitors from the NIKOM library to identify new candidates that inhibit SIRT1 activity (Supplementary Figs. 4–8). Consequently, we identified four compounds, namely dioscin (04F01), xanthoangelol (04G01), ent-7b-hydroxy-15-oxokaur-16-en-18-ylacetate (05C03) and mulberrofuran C (05C09), that inhibited SIRT1 activity over 80% (red color) and then the top five compounds, namely (*E*)-5-hydroxy-3-7-methoxy-(2’-hydroxybezylidene)-4-chromanome (05F09), kurarinone (06A06), sophoraflavanone G (06B03, vexibinol), frugoside (09G08) and KWN-C (10B08), that reduced SIRT1 activity less than 80% (blue color) (Supplementary Table 1). Next, H358 and H460 cells were dose-dependently treated with eight commercially available compounds except for ent-7b-hydroxy-15-oxokaur-16-en-18-ylacetate. 05C09, 06A06, 09G08 and 10B08 strongly reduced SIRT1 activity more than 04F01, 04G01, 05F09 and 06B03 (Supplementary Fig. 9a,b). These former four compounds were subsequently screened to confirm their minimum toxicity levels, because the inhibitor of SIRT1 activity was used in combination treatments with CP and MTA. Both 05C09 and 06A06 showed a compound, dose-dependent toxicity, while 09G08 inhibited cell viability over 80% irrespective of dose used. Only 10B08, KWN-C, showed minimal toxicity (Supplementary Fig. 9c). In addition, KWN-C did not decrease SIRT1 protein levels in a dose-dependent manner (Supplementary Fig. 10). Based on these results, KWN-C was selected from all natural product compounds evaluated as the SIRT1 activity inhibitor with minimal toxicity.

### TGF-β1–Smad2/3–JNK signaling-mediated SIRT1 activity is suppressed by KWN-C

**Fig. 4:**
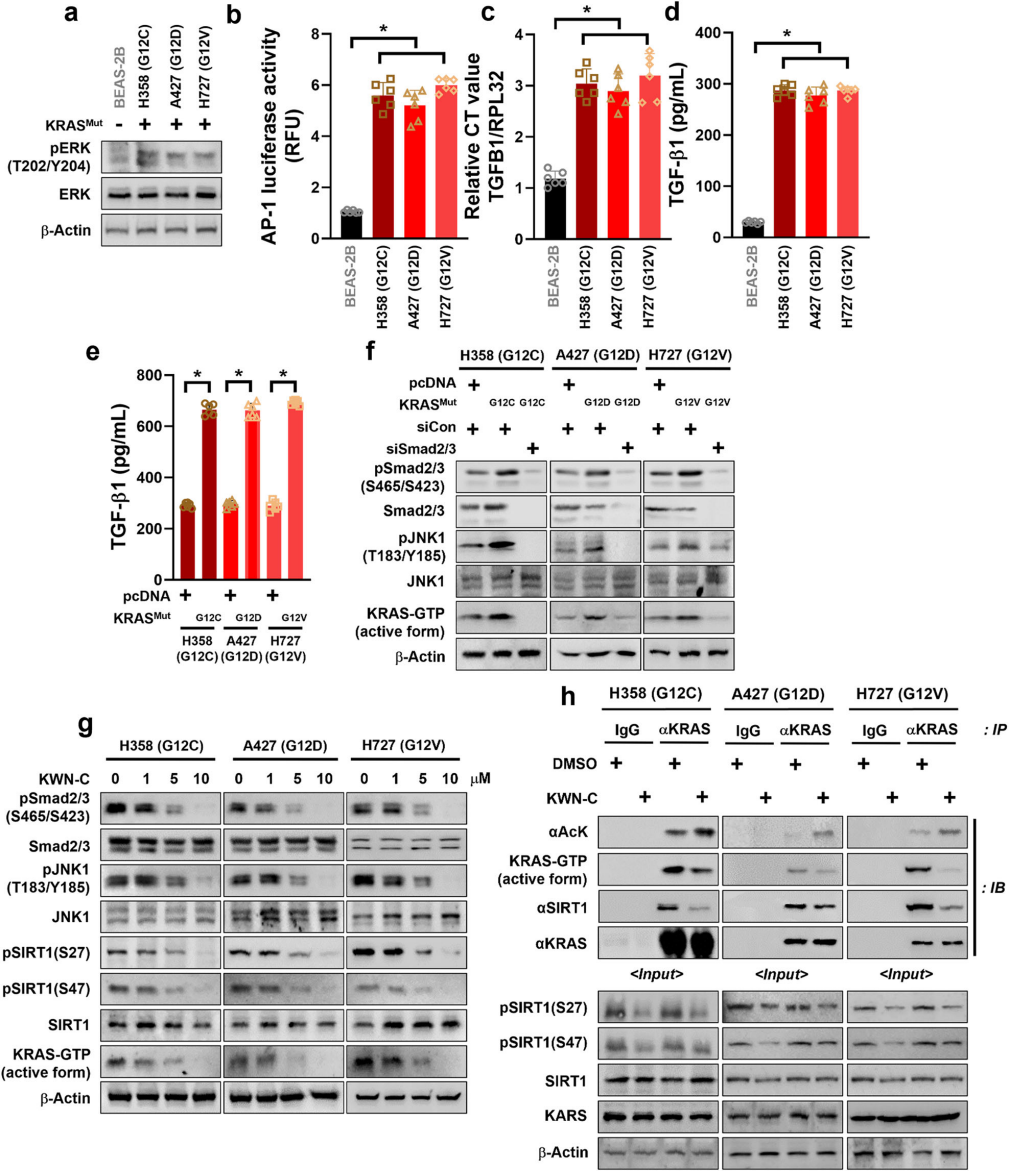
SIRT1 activity in KRAS^Mut^ cells is dependent on MEK–ERK–AP-1 pathway-mediated TGF-β1–Smad2/3–JNK signaling, which is blocked by KWN-C. **a** Normal human bronchial epithelial cell (BEAS-2B) and KRAS^Mut^ cell lines (H358, A427 and H727) were collected with lysis buffer and subjected to western blotting with anti-pERK, ERK and β-actin antibodies. **b** A luciferase assay was performed to assess the AP-1-mediated transcriptional regulatory activity with cell lysates in Fig. 4a. Student’s *t*-test, mean ± s.d.; *n* = 6, *P < 0.05. **c** TGFB1 mRNA expression was measured by RT–qPCR with same cell lines as in **a**. *RPL32* was used as internal control and for normalization. Student’s *t*-test, mean ± s.d.; *n* = 6, *P < 0.05. **d** The medium of four cell lines were changed by FBS-free medium before cell collection at 24 h. Conditioned medium was collected and concentrated using an Amicon Ultra-15 tube, and total TGF-β1 levels were measured by ELISA. **e** KRAS^Mut^ cell lines (H358, A427 and H727) were transfected with *pcDNA*, *KRAS G12C*, *G12D* and *G12V* plasmids (2 μg). TGF-β1 levels were measured under the same method as in **d**. **f** H358, A427 and H727 cells were transplanted with *pcDNA*, *KRAS G12C*, *G12D* and *G12V* plasmids, siCon and siSmad2/3 (80 nM) for 48 h, and then the activity of Smad2/3, JNK1 and KRAS was measured. **g** The cell lysates of each different KRAS^Mut^ cell lines (H358, A427 and H727) under KWN-C with indicated dosage for 24 h were transferred by immunoblotting assay with anti-pSmad2/3, anti-Smad2/3, pJNK1, JNK1, anti-pSIRT1^Ser27^, pSIRT1^Ser47^, SIRT1, KRAS–GTP-bound and β-actin antibodies. **h**, H358, A427 and H460 cells were treated with DMSO or KWN-C (10 μM), and cell extracts were then immunoprecipitated using immunoglobulin G, anti-KRAS, and RAF-1 agarose bead antibodies. Immunoblotting was performed using anti-acetyl, anti-KRAS–GTP-bound, anti-SIRT1, anti-KRAS and β-actin antibodies.

To delineate the exact mechanism by which KWN-C suppresses SIRT1 activity, we investigated similar chemical compounds already known their target to KWN-C via https://mcule.com/ (Mcule). We identified 182 compounds with a Tanimoto coefficient score above 7.0, and subsequently searched for compounds associated with the JNK pathway due to its role in JNK1-induced SIRT1 activity in KRAS^Mut^ lung cancer. We identified Dalbergioidin (with a Tanimoto coefficient score of 0.715), a well-known anthocyanin, which ameliorates doxorubicin-induced renal fibrosis by suppressing the TGF-β signaling pathway^33^. Therefore, we investigated the association of KWN-C with the induction signaling, TGF-β1–Smad2/3–JNK, required to activate SIRT1. First, we addressed the mechanisms through which KRAS^Mut^ enhances the expression of TGF-β1 cytokines. Because oncogenic KRAS leads to the activation of the MEK–ERK pathway, we confirmed that this pathway was activated in mutated KRAS cells. Our results showed that the pERK levels in KRAS^Mut^ cells were higher than those in BEAS-2B cells (Fig. 4a). Given that ERK can modulate gene expression by activating several transcription factors, such as AP-1, and that the human promoters of TGF-β1 contain several binding sites for AP-1^34^, we hypothesized that AP-1 could be involved in KRAS-induced transcription of TGF-β1. To explore this hypothesis, we assessed AP-1 luciferase activity. As expected, AP-1 transcription activity in KRAS^Mut^ cells was approximately sixfold higher than that in BEAS-2B cells (Fig. 4b), suggesting that AP-1 may play a role in TGF-β1 upregulation. Taken together, these data demonstrate that oncogenic KRAS induced the expression of TGF-β1 through activation of the MEK–ERK–AP-1 pathway. Moreover, TGF-β1 mRNA levels in KRAS^Mut^ cells were approximately threefold higher than those in nontumorigenic epithelial cells (Fig. 4c). ELISA was then performed to quantify the production of TGF-β1 ligands secreted in an autocrine manner in the culture medium. KRAS^Mut^ cells were found to secrete significantly higher amounts of TGF-β1 compared with BEAS-2B cells (Fig. 4d) To confirm our previous results, the same experiments were performed after overexpression with three different types of KRAS^Mut^ plasmid in normal bronchial epithelial cells, BEAS-2B cells. Our findings demonstrated that the expression of KRAS^Mut^ plasmids increased the activity of ERK–AP-1 signaling even in nontumorigenic epithelial cells, which in turn increased the mRNA levels and protein release of TGF-β1 (Supplementary Fig. 11a–d). These results indicated that the higher levels of TGF-β1 secretion observed in KRAS ^Mut^ cells were caused by an increase in TGF-β1 mRNA expression by the AP-1 transcription factor.

Next, we investigated whether ERK–AP-1 pathway-induced TGF-β1 activates the Smad2/3–JNK1 pathway through an autocrine manner because the Smad2/3–JNK1 pathway is a downstream effector of the TGF-β pathway. We first identified TGF-β1 secretion from KRAS^Mut^ cells after pcDNA and KRAS^Mut^ plasmid expression. TGF-β1 secretion in KRAS^Mut^ plasmid-expressing cells was more than twofold higher compared with cells harboring pcDNA (Fig. 4e). Next, we observed substantial phosphorylation of Smad2/3 in KRAS^Mut^ cells expressing KRAS^Mut^ plasmids. The subsequent Smad2/3-mediated JNK1 activity was substantially enhanced in KRAS^Mut^ cells, which also increased KRAS^Mut^ activity. However, Smad2/3-specific siRNA transfected cells did not demonstrate any JNK1 and KRAS^Mut^ activation (Fig. 4f). These results indicate that the KRAS^Mut^-induced TGF-β1–Smad2/3 pathway activates JNK1 in KRAS^Mut^ cells. To confirm the involvement of the TGF-β1–Smad2/3 pathway in the mechanism involved in the inhibition of SIRT1 activity in KRAS^Mut^ cells by KWN-C, KWN-C was treated in a dose-dependent manner. Our findings showed that KWN-C suppressed pSmad2/3–JNK1 levels, which subsequently abrogated the phosphorylation of SIRT1^S27^ and SIRT1^S47^ (Fig. 4g). Consequently, KWN-C treatment decreased SRIT1 activity, which in turn increased KRAS acetylation and decreased KRAS activity. To identity whether KWN-C decreases KRAS^Mut^ activity via reducing SIRT1 activity and enhancing the acetylation of KRAS^Mut^, an immunoprecipitation assay was performed under KWN-C treatment in KRAS^Mut^ cells. As expected, KWN-C treatment of cell lysates resulted in increased acetylation of KRAS^Mut^ and reduced KRAS^Mut^ activity by suppressing SIRT1 activity (Fig. 4h). These results suggests that the MEK–ERK–AP-1 pathway is prominently involved in the enhancement of TGF-β1 secretion in KRAS^Mut^ cells, subsequently increasing TGF-β1–Smad2/3–JNK signaling through an autocrine manner. However, KWN-C substantially decreased SIRT1 enzymatic activity owing to the suppression in TGF-β1–Smad2/3 signaling and subsequent inhibition of KRAS^Mut^ activity. Moreover, as a SIRT1 activity inhibitor, KWN-C could be a useful therapeutic candidate with first-line chemotherapy for patients with KRAS^Mut^ NSCLC.

### KWN-C cotreatment enhances the efficacy of CP and MTA in KRAS^Mut^ NSCLC cells

**Fig. 5:**
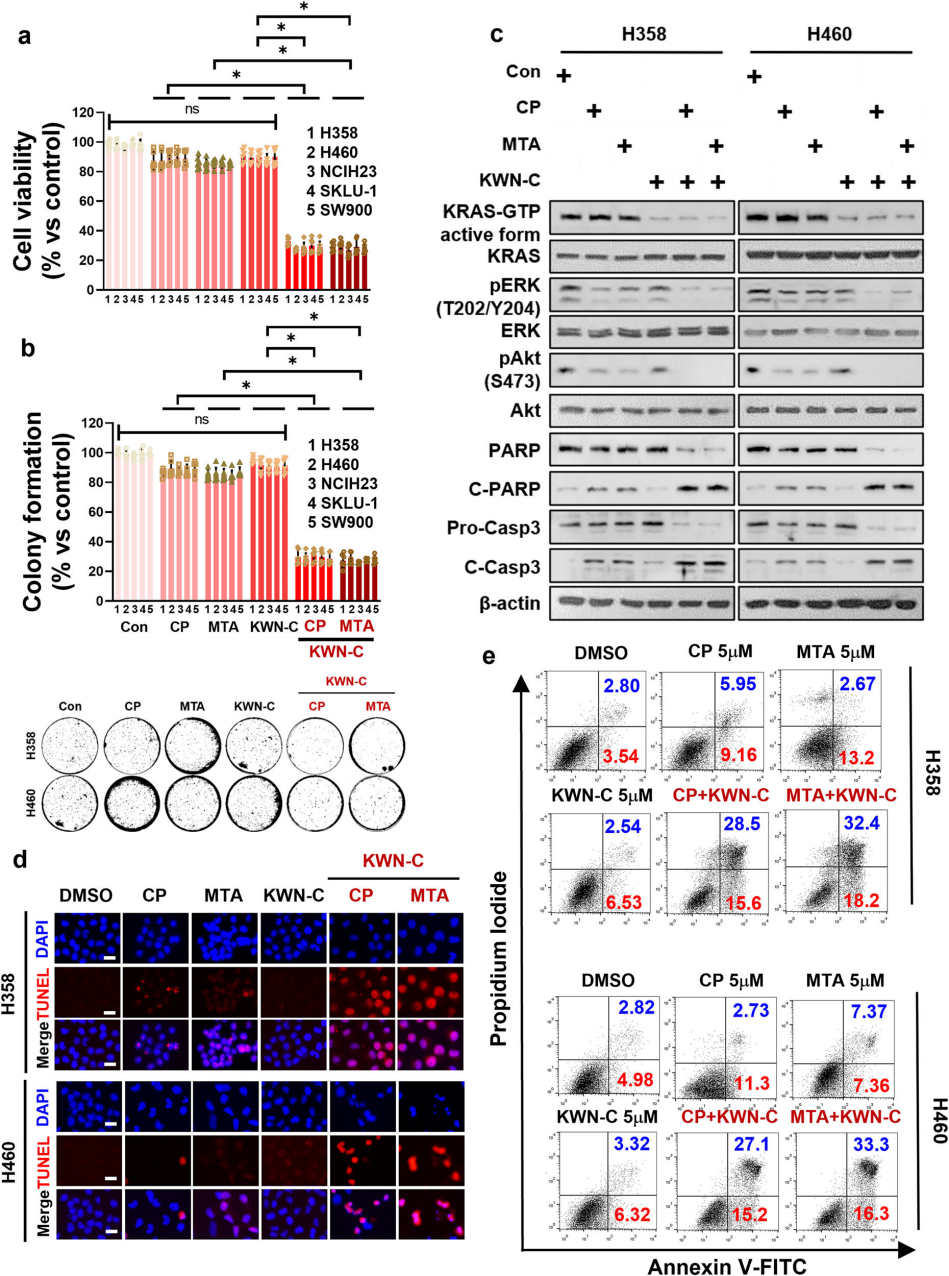
SIRT1 inhibitor synergistically decreased KRAS^Mut^ lung cancer proliferation combined with CP and MTA. **a** H358, H460, NCIH23, SKLU-1 and SW900 cells were treated with CP (H358 1 μM, H460, NCIH23 and SKLU-1 5 μM), MTA (H358 10 μM, H460, NCIH23 and SKLU-1 5 μM) and/or KWN-C (10 μM), and cell proliferation was measured by the MTS assay 3 days after drug treatment. Student’s *t*-test, mean ± s.d.; *n* = 6, *P < 0.05. **b** Top: KRAS^Mut^ cells were seeded with 0.5% top agar and cultured in a mixture of fresh medium with drugs as described in Supplementary Fig. 5a. Cell colonies were stained with crystal violet and counted per 3.8 cm^2^. Bottom: representative colony images are shown. Student’s *t*-test, mean ± s.d.; *n* = 6, *P < 0.05. **c** H358 and H460 cells were treated with CP (H358 1 μM and H460 5 μM), MTA (H358 10 μM and H460 5 μM) and/or KWN-C (10 μM). Cell lysates from drug-treated cells were incubated with Raf-1–RBD to pull down KRAS–GTP (the active form of KRAS), followed by western blotting with an anti-KRAS antibody. Expression levels of KRAS, pERK, ERK, pAkt, Akt, PARP, cleaved PARP, pro-caspase-3, cleaved-caspase-3 and β-actin in total lysates were analyzed by western blotting. **d** H358 and H460 cells treated as in **a** were assessed for apoptosis by TUNEL assay (middle row), and their nuclei were stained with DAPI (top row; scale bar, 25 μm). All figures are representative of at least three separate experiments. **e** H358 and H460 cells treated as in **a** were stained with Annexin V/PI staining for apoptosis using flow cytometric analysis. Representative flow cytometry plots. All figures are representative of at least three separate experiments.

Patients with lung cancer harboring *KRAS* mutations have shown to be resistant to anticancer drugs of CP and MTA^35^, and as shown in Fig. 2a, the control group of H358 and H460 cells treated with CP and MTA showed chemoresistance. To test whether the SIRT1 activity inhibitor KWN-C showed synergistic anti-KRAS^Mut^ cancer effects under combination treatment with CP or MTA, cell viability and colony formation number were measured in H358, H460, NCIH23, SKLU-1, and SW900 cells. These KRAS^Mut^ cells showed resistance under CP and MTA single treatment, whereas the combination KWN-C and CP or MTA synergistically decreased cell viability and colony formation number (Fig. 5a,b). However, single treatments with CP and MTA reduced cell viability and colony formation in EGFR^Mut^ cells (H1650, H1975, H827 and H2279) and KRAS^WT^ and EGFR^WT^ cells (H1666, H322M, H522 and Calu-3) by over 40%. The combination treatment with KWN-C did not further decrease cell viability or colony formation compared with the single treatments (Supplementary Figs. 12 and 13). Based on these results, it can be inferred that KWN-C specifically sensitized the anticancer effects of CP and MTA in KRAS^Mut^ lung cancer by reducing the activity of KRAS^Mut^ and SIRT1. To investigate why KWN-C synergistically inhibited the proliferation of KRAS^Mut^ NSCLC cells only, we performed western blot, TUNEL staining and PI and annexin V staining analysis of both apoptotic and survival markers after treatment with CP, MTA and/or KWN-C. When KWN-C was treated with CP or MTA, the apoptotic markers, cleaved PARP and cleaved- caspase 3, were robustly increased and decreased, respectively, while the survival markers, pERK and pAkt, were strongly decreased compared with each single treatment or nontreatment in western blot (Fig. 5c). Likewise, the combination treatments of CP, MTA and KWN-C showed higher levels of TUNEL-positive cells and double-positive cells of PI and annexin V staining compared with each single treatment or nontreatment (Fig. 5d,e). The double-positive cells of PI and annexin V staining (blue color) and the single-positive cells of annexin V (red color) are shown in Supplementary Fig. 14. The synergistic anticancer effects in the combination treatment group were responsible for reducing KRAS^Mut^ activity and downsignaling pERK and pAkt by KWN-C (Fig. 5c). Collectively, our results revealed that natural product compounds of the SIRT1 activity inhibitor KWN-C have a potent role as an adjuvant therapy to overcome KRAS^Mut^ chemoresistance.

### Determination of the optional therapeutic index of KWN-C for in vivo experimentation

**Fig. 6:**
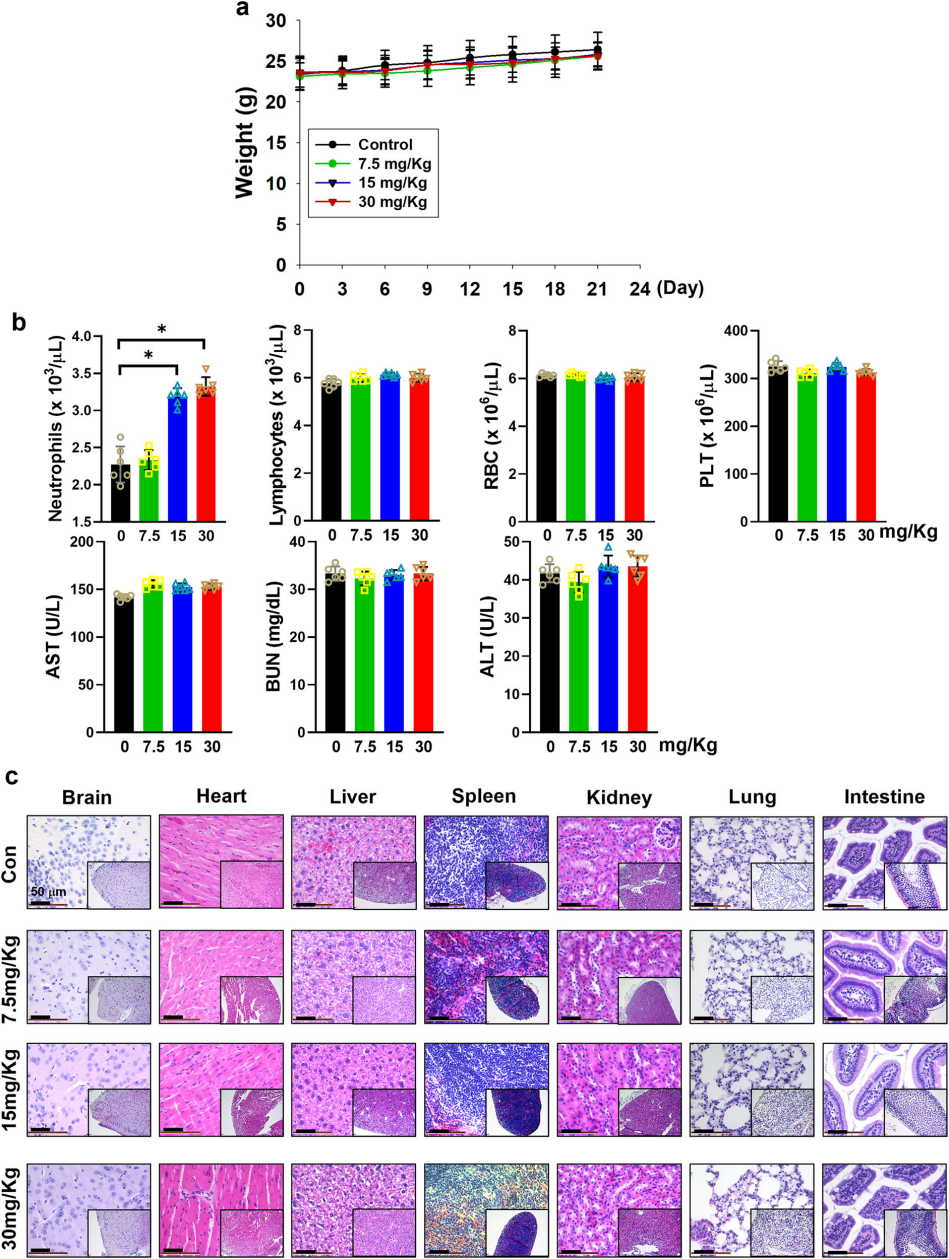
Evaluation of in vivo toxicity of SIRT1 activity inhibitor KWN-C from natural product compound screening. **a** Nu/Nu nude mice were treated with increasing doses of KWN-C (0, 7.5, 15 and 30 mg/kg per day, i.p.) for 21 days (*n* = 6 mice per group). Body weight of mice was measured every 3 days during treatment with various doses of KWN-C. **b** Blood analysis of mice after treatment with various doses of KWN-C for 21 days. Student’s *t*-test, mean ± s.e.m.; *n* = 6, *P < 0.05. **c** H&E staining histology of brain, heart, liver, spleen, kidney, lung and intestine from mice after treatment with KWN-C dose dependently for 21 days. Scale bar, 50 μm.

To define the appropriate dose of KWN-C for in vivo experimentation, we first determined the standard single-dose maximum tolerated dose (MTD) following Zhang et al.^36^. Nu/Nu nude mice were treated with a single dose of 150, 300 and 400 mg/kg i.p., followed by toxicity observations. Treatment of mice with a single dose of 150 and 300 mg/kg i.p. did not induce any weight loss or other toxicities, including hematologic disorders, or liver and kidney function abnormalities. However, a single dose of 400 mg/kg resulted in the death of mice within 3 days. Furthermore, ALT, AST and BUN were significantly elevated (Supplementary Fig. 15). Based on these findings, the cause of death was mainly due to liver and kidney damage at a single dose of 400 mg/kg. Thus, the single dose MTD of KWN-C ranges from 300 to 400 mg/kg. Generally, 10% of a single-dose MTD can usually be considered the maximum therapeutic dose for continuous treatment^37^. Therefore, doses below 30 mg/kg per day were considered to be relatively safe, and three daily safe doses, that is, 7.5, 15 and 30 mg/kg/day, were selected, which were well tolerated without notable toxicity. Our findings showed that there was no weight loss (Fig. 6a), and blood tests (WBC, RBC and PLT) for bone marrow, BUN for kidney and ALT/AST for liver functions were in the normal range (Fig. 6b). Histopathology of collected normal tissues of brain, heart, liver, spleen, kidney, lung and intestines revealed no evidence of normal tissue toxicities after treatment with doses of ~7.5–30 mg/kg per day (Fig. 6c). These findings indicate that doses between 7.5 and 30 mg/kg per day provide the optional therapeutic index of KWN-C for in vivo studies.

### Combination treatment with CP, MTA and KWN-C synergistically reduces tumor burden in lung orthotropic models bearing *KRAS*^*G12C*^

**Fig. 7:**
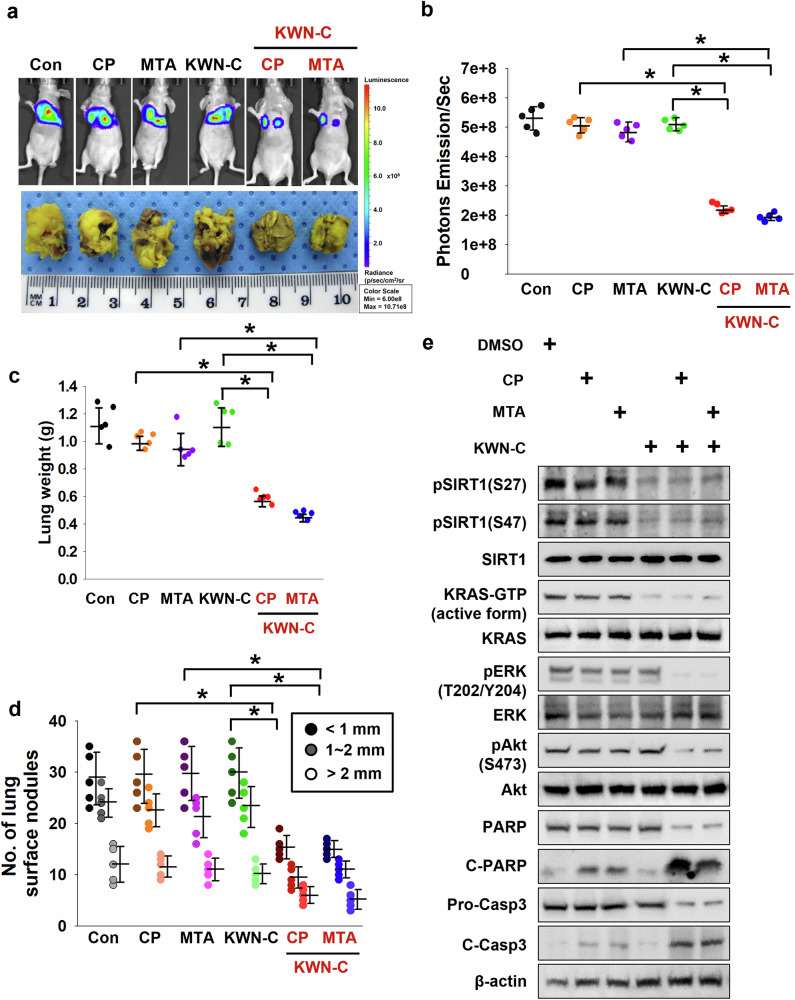
The combination CP, MTA and SIRT1 activity inhibitor synergistically decreases KRAS^Mut^ lung orthotopic tumorigenesis. **a** H358 cells harboring stably expressed luciferase plasmid were intratracheally injected into nude mice (1 × 10^6^ cells per mouse). Top: representative bioluminescence images 2 months after the injection. The mice were euthanized 2 months after the injection, and lungs were excised and stained with Bouin’s fixative. Bottom: the lung tumor images. Therapeutic candidates were treated with CP (5 mg/kg per day, i.p.), MTA (150 mg/kg twice a week, i.p.) and/or KWN-C (30 mg/kg per day, i.p.). **b** The photon emission values represent the mean ± s.e.m. of the indicated number of mice. Student’s *t*-test, mean ± s.e.m.; *n* = 5, *P < 0.05. **c** The lung tumor weight from combination CP, MTA and/or KWN-C-treated mice was measured and compared with nontreatment, each single treatment and combined treatment. Student’s *t*-test, mean ± s.e.m.; *n* = 5, *P < 0.05. **d** The number of colonies formed in the lungs were measured under microscopy under the same conditions as in **c**. Student’s *t*-test, mean ± s.e.m.; *n* = 5, *P < 0.05. **e** KRAS–GTP (active form of KRAS) was pulled down by Raf-1–RBD from tumor tissue lysates, followed by western blot using KRAS antibody. Expression levels of anti-pSIRT1^S27^, pSIRT1^S47^, SIRT1, KRAS–GTP-bound, KRAS, pERK, ERK pAkt, Akt, PARP, cleaved PARP, pro-caspase-3, cleaved-caspase-3 and β-actin were analyzed by western blot in tumor tissues.

To confirm the in vivo benefits of KWN-C and/or CP and MTA induced by KRAS^Mut^, H358 cells stably expressing luciferase plasmid were inoculated via intratracheal injection. H358 cells under either CP or MTA single treatment showed a markedly aggressive behavior in luciferase and Bouin’s solution staining lung images, a behavior that was significantly reduced following a combination treatment with KWN-C (Fig. 7a,b). Our findings showed that both lung weight and the number of colonies in the lungs were significantly lower in the combination treatment group than those in each single or nontreatment groups (Fig. 7c,d). To assess whether KWN-C induced suppression of tumor growth via apoptosis in vivo, representative samples from collected tumor tissues were analyzed by immunoblotting. In accordance with the in vitro results, the samples treated with KWN-C showed decreased phosphorylation of SIRT1^S27^ and SIRT1^S47^, resulting in reduced KRAS^Mut^ activity in the Raf-1–RBD pulldown experiments for KRAS activity. The combination of CP or MTA with KWN-C decreased or increased the levels of pERK and pAkt, respectively, while simultaneously increasing apoptotic markers such as cleaved-PARP and cleaved-caspase 3, compared with the single treatments or control samples (Fig. 7e).

### KWN-C potently inhibits tumor growth and prolongs survival of mice with genetically engineered G12D mutant KRAS-driven lung cancer

**Fig. 8:**
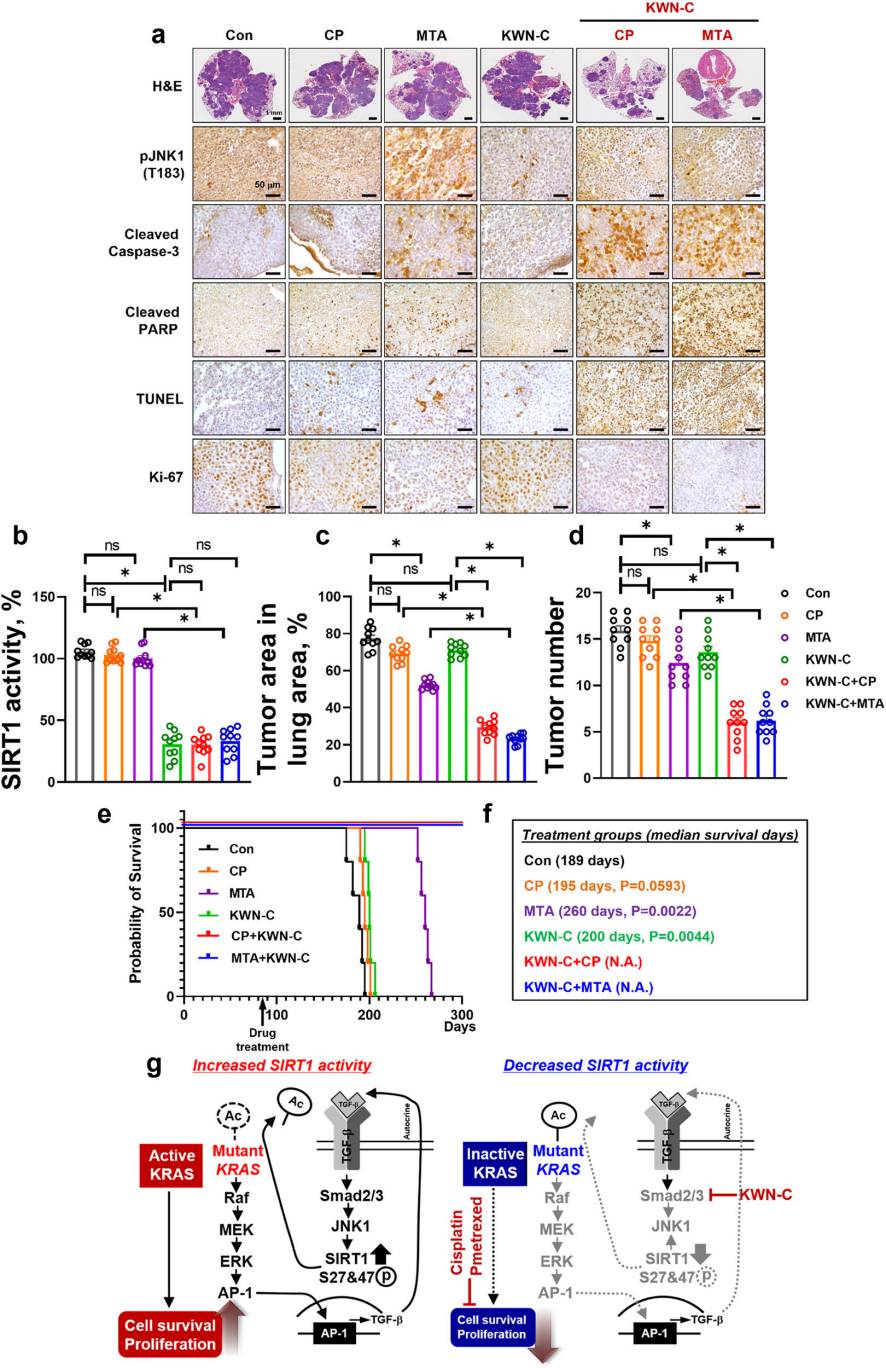
SIRT1 activity inhibitor synergistically sensitizes the anticancer effect of CP and MTA in a KRAS^G12D^ spontaneous lung tumor model. **a** After administration of adenovirus Cre recombinase in KRAS^G12D^ mice for 10 weeks, mice were treated using the same method as in Fig. 7. Representative H&E staining images and pJNK1, cleaved caspase-3, cleaved PARP, TUNEL and Ki-67 were analyzed by immunohistochemical staining in tumor tissues at the end of experiments. **b** Tumor tissue from each drug-treated group was collected with lysis buffer, and SIRT1 activity was measured with cell lysates. Student’s *t*-test, mean ± s.e.m.; *n* = 10, *P < 0.05. **c** Tumor area was quantified using ImageJ software. Student’s *t*-test, mean ± s.e.m.; *n* = 10, *P < 0.05. **d** Tumor numbers per lung area were counted under the microscope in specimens collected from mice treated with drugs. Student’s *t*-test, mean ± s.e.m.; *n* = 10, *P < 0.05*.* **e** Survival rates of mice treated with drugs (log-rank test). **f** Median survival days and *P* values were calculated using the log-rank test and the Gehan–Breslow–Wilcoxon test, respectively, based on Student’s *t*-test. **g** A schematic overview of the mechanism of enhanced SIRT1 activity in KRAS^Mut^ lung cancer and definition of a rational combination strategy between SIRT1 activity inhibitor and conventional chemotherapy.

KRAS mutations are common genetic alterations in NSCLC and contribute to the resistance of lung cancer to conventional chemotherapy^38–40^. To assess whether KWN-C could sensitize antitumor activity of CP and MTA against KRAS^G12D^-driven lung cancer in genetically engineered mouse models, KWN-C (20 mg/kg per day) or a vehicle control was administered i.p. starting at 12 weeks after Ade-Cre delivery as previously suggested^23^. After treatment for approximately 6.7 months, mice were euthanized for analysis of SIRT1 activity, tumor area and tumor number. H&E staining results showed that treatment with KWN-C combined with CP or MTA resulted in significant reduction of tumor area and tumor number in KRAS^G12D^ mice (Fig. 8a,c,d). The synergistic anticancer effects in the combination treatment groups were attributed to the fact that KWN-C reduced JNK activity, which in turn reduced SIRT1 activity. Therefore, the reduction in KRAS^Mut^ activity facilitates the anticancer effects of CP and MTA, leading to an increase in cleaved-caspase 3, cleaved-PARP and TUNEL-positive cells, while decreasing Ki-67 expression compared with each single treatment and nontreatment group (Fig. 8a,b). In addition to a reduced tumor burden in both combination treatment groups, deaths were noted in the groups treated with CP and MTA under KWN-C; however, the median survival time of control in CP, MTA and KWN-C was less than 260 days (Fig. 8e,f). These results strongly suggest that decreasing SIRT1 activity in KRSA^Mut^ lung cancer inhibited tumorigenesis in KRAS^Mut^ engineered mouse models, indicating that the combination of KWN-C with established chemotherapy represents a potential therapeutic strategy in *KRAS*^*Mut*^ lung cancer.

## Discussion

KRAS plays a pivotal role as a proto-oncogene in signal transduction of cell proliferation; however, KRAS^Mut^ is closely related to tumor initiation and development. Thus, it is essential to develop a successful targeting therapy for KRAS^Mut^, thereby leading to the introduction of a novel platform for targeted KRAS^Mut^ therapy. KRAS^Mut^ is notoriously challenging to target. It is also undruggable owing to its smooth, spheric structural biology and a lack of drug-binding pockets, thereby limiting potential onco-therapeutic approaches. Consequently, researchers shifted to other important molecules in the KRAS signaling pathway, such as RAF, ERK and MEK. However, there has been no notable success in KRAS-driven tumors, which partly explains the poor efficacy of targeting the KRAS signaling pathway. Therefore, alternative strategies aimed at inhibiting KRAS protein expression with antisense oligonucleotides or RNA interference were used. Unfortunately, both the antisense approach and RNA interference have been limited by the ability to safely and effectively deliver antisense oligonucleotides and siRNAs of KRAS.

KRAS acts as a switch for key sensors that trigger the activation of multiple signaling molecules, enabling the transmission of signals from the cell surface to the nucleus. This process affects a range of essential cellular functions, including cell differentiation, growth, chemotaxis and apoptosis. Activation of the KRAS signaling pathway is a multistep process that requires proper KRAS posttranslation, plasma membrane-localization and interaction with effector proteins, mechanistic processes that can be exploited for the development of KRAS-signaling-targeted therapies. Both processes are regulated by KRAS posttranslational modification consisting of prenylation, proteolytic cleavage, methylation and palmitoylation^25^. Inhibiting any of these steps could theoretically decrease KRAS activity, interfering in its association with the plasma membrane and regulating its interaction with effectors and protein stability. Among these, prenylation is the rate-limiting step in the posttranslational modification of KRAS proteins. The CAAX motif within KRAS serves as the substrate for farnesyltransferase, which weakens the protein’s affinity for the plasma membrane. FTIs were the first class of anticancer agents identified to target KRAS prenylation, and a number of FTIs have been tested in clinical trials. Although FTIs were initially designed to target KRAS, they failed to represent a meaningful strategy to target KRAS for cancer therapy, because a number of important signaling molecules are farnesylated, including Rho-B, Rho-E, Rap2A, PTP-CAAX, CENP, Lamin and HDJ2^6^. Consequently, there is a growing need to develop selective inhibitors that target specific KRAS^Mut^, to effectively inhibit the distinct functions of different KRAS^Mut^, aligning with the goals of precision oncology. Finally, four decades of research culminated in the first major breakthrough in the race to target KRAS-driven cancers. In 2013, a seminal breakthrough by the Shokat lab showed that the activated KRAS isoenzyme, caused by the KRAS^G12C^ gene, can be directly inhibited via a newly identified switch II pocket^41^. In vitro, preclinical and clinical trial data demonstrated its antitumor activity and clinical efficacy. However, similar to other targeted therapies, these KRAS^G12C^ inhibitors are also plagued by both intrinsic and acquired resistance mechanisms, which limit their efficacy and duration of response in patients with KRAS^G12C^ lung cancer^42–44^. In addition, KRAS^G12C^ inhibitors are dependent on the covalent inhibition of cysteine and high GTPase activity of KRAS^G12C^, which is not fully present in other KRAS^Mut^ types^45^. Therefore, novel strategies were necessary for targeting or decreasing other common KRAS^Mut^ activity, such as KRAS^G12D^ and KRAS^G12V^.

KRAS is small and has a considerably flexible C-terminal hypervariable region that is posttranslationally modified to regulate its membrane localization and activity. In our study, we found that KRAS^Mut^ specifically upregulates SIRT1 expression and activity, forming a positive feedback loop that enhances its own signaling potency. As shown in Fig. 1, SIRT1 mRNA and protein levels were significantly higher in KRAS^Mut^ NSCLC cell lines compared with KRAS^WT^ or EGFR^Mut^ cell lines, and this was further validated in primary lung tumors from LSL-Kras^G12D^ transgenic mice. Overexpression of KRAS^G12D^ or KRAS^WT^ led to increased SIRT1 protein levels, but KRAS^Mut^ induced greater SIRT1 activity at lower plasmid doses, indicating a mutation-specific regulatory effect, especially under nonsaturating conditions (Supplementary Fig. 2). Co-immunoprecipitation assays also demonstrated that KRAS^Mut^ exhibits stronger binding to SIRT1 than KRAS^WT^ (Supplementary Fig. 3), suggesting a tighter functional interaction between oncogenic KRAS and this deacetylase. Importantly, this interaction has functional consequences: SIRT1 deacetylates KRAS at lysine 104, a modification previously shown to inhibit GTP loading. Thus, SIRT1-mediated deacetylation removes this inhibitory signal, increasing KRAS^Mut^ activity. These findings propose that, in KRAS^Mut^ NSCLC, SIRT1 is not only upregulated but actively enhances KRAS oncogenic signaling through posttranslational modulation—forming a self-reinforcing loop unique to the KRAS-mutant context.

In addition, KRAS is often considered an undruggable target due to the intrinsic characteristics of its protein structure. Consequently, our group suggested an indirect strategy of reducing KRAS^Mut^ activity by targeting SIRT1. The class III histone deacetylase SIRT1 expression was upregulated by the KRAS^Mut^–Raf–MEK–c-Myc axis in KRAS^Mut^ lung cancer, while KRAS^Mut^-induced SIRT1 increased KRAS^Mut^ activity via stable deacetylases of KRAS^Mut^ at lysine 104. SIRT1 K/D also increased KRAS^Mut^ acetylation and then decreased KRAS^Mut^ activity. Our findings indicate that SIRT1 targeting has great advantages for KRAS^Mut^ lung cancer. First, we focused on the minimum cytotoxic capacity of targeting SIRT1 by itself. When combining the SIRT1 activity inhibitor with existing anticancer drugs as an adjuvant agent, the combination therapy showed the greatest synergistic anticancer effects. In this regard, although a single treatment with the SIRT1 activity inhibitor did not demonstrate any antitumor properties against KRAS^Mut^ lung cancers by itself, combination of the SIRT1 activity inhibitor with chemotherapy showed a higher cytotoxic effect than that induced by chemotherapy alone, reversing the chemoresistance conferred by the KRAS^Mut^–SIRT1 axis in both in vitro and in vivo models. Therefore, we concluded that the SIRT1 activity inhibitor is a good candidate combination with current treatments for patients with KRAS^Mut^ lung cancer. As mentioned in this study, KWN-C is a promising candidate for combination therapy with existing anticancer drugs and natural products. SIRT1 inhibition enhances the sensitivity of cancer cells to chemotherapy, thereby boosting its anticancer effects. Second, the three dominant types of KRAS^Mut^, namely G12C, G12D and G12V, are subject to KRAS^Mut^ activity regulation by SIRT1. These three types of KRAS^Mut^ are also prevalent in colorectal and prostate cancer as well as lung cancer. Moreover, the strategy of combining an SIRT1 activity inhibitor with existing chemotherapy could enhance anticancer effects across most types of KRAS^Mut^. Provided that this hypothesis is valid, our strategy is extremely economical and efficient for patients with KRAS^Mut^ colorectal, prostate and lung cancer.

Notably, our findings demonstrated that SIRT1 activity in KRAS^Mut^ cells is higher compared with normal cells, EGFR^Mut^-positive cancer cells and KRAS^Mut^/EGFR^Mut^-negative cancer cells, as indicated by elevated SIRT1 protein expression (Fig. 3a). There are many different stimuli and conditions that can be used to increase SIRT1 activity. For example, SIRT1 activity can be directly regulated by substrate availability, posttranslational modifications, interacting protein partners or small-molecule activators or repressors^46^. Among these, we focused on posttranslational modifications as regulators of SIRT1 activity in KRAS^Mut^ lung cancer, emphasizing the role of phosphorylation. This was based on the observation that JNK1 phosphorylation levels were significantly higher in KRAS^Mut^ lung cancer cells compared with normal bronchial epithelial cells, mediated by MKK4 and MKK7 (Fig. 3b). SIRT1 has at least 20 serine/threonine phosphorylation sites in its N- and C-terminal domains^47^. JNK1 is capable of phosphorylating SIRT1 at Ser27 and Ser47 (mouse 46), and at Thr530 in colorectal cancer and mouse myoblasts. Intriguingly, these phosphorylations appear to increase the deacetylase activity of SIRT1 toward one of its substrates, histone H3. By contrast, they have no effect toward other substrates, such as p53 in colorectal cancer and mouse myoblasts^30,47,48^. In KRAS^Mut^ lung cancer cells, the phosphorylation of Ser27, Ser47 and Thr530 on pSIRT1 depended on JNK1 activators and inhibitors. Notably, phosphorylation at Ser27 and Ser47 of pSIRT1 was involved in the deacetylation of KRAS^Mut^. In the current study, substituting serine 27 and 47 with alanine resulted in greater activity and deacetylation of KRAS^Mut^ compared with Thr530 on pSIRT1 (Fig. 3f). Moreover, these results suggest that JNK1-mediated Ser27 and Ser47 of pSIRT1 enhance KRAS^Mut^ activity through an increase in the catalytic activity of SIRT1. The JNK1–SIRT1 pathway provides a new mechanism for its interaction with KRAS^Mut^. In addition, phosphorylation of human SIRT1 at serine 47 mediated by JNK1 has been showed to be related to its ubiquitin-dependent degradation^49^. However, our results showed that JNK1-mediated Ser47 of pSIRT1 did not degrade SIRT1 in KRAS^Mut^ lung cancer cells (Fig. 3). This probably indicates that SIRT1 phosphorylation facilitates the recruitment of additional binding partners, such as USP7 or USP22, which act as endogenous stabilizers of SIRT1 by preventing its ubiquitination. Alternatively, a phosphorylation-induced conformational change of SIRT1 could block access to E3 ubiquitin ligase(s), thereby maintaining its stability^50^.

It is widely known that TGF-β1 plays a two-sided role in lung tumorigenesis^51^. In the early stages of lung tumor development, TGF-β1 acts as a tumor suppressor, inhibiting cell cycle progression and proliferation, preventing cellular immortalization and promoting cellular differentiation or apoptosis. In the later stages of lung tumorigenesis, TGF-β1 acts as a tumor stimulator, promoting cellular changes associated with migration, invasion, metastasis, immunosuppression, angiogenesis, myofibroblast generation, interactions between cancer cells and extracellular matrix, and epithelial–mesenchymal transition^52^. These complex interactions of the tumor stimulator roles of TGF-β1 may be a key factor in KRAS^Mut^ lung cancer resistance to chemotherapy. A number of recent studies have shown that activation of the TGF-β1 signaling pathway is associated with drug resistance in KRAS^Mut^ cancers, including NSCLC^53^, colorectal cancer^54^ and prostate cancer^55^. Moreover, high levels of TGF-β1 in patients with NSCLC resulted in poor prognosis^56^. In our study, KRAS^Mut^ activation increased pERK level and activated the AP-1 transcription factor. Our findings confirmed that AP-1 enhanced KRAS^Mut^-induced transcription, protein levels and secretion of TGF-β1. In addition, the introduction of the KRAS^Mut^ genes into tumor cells with KRAS^WT^ significantly enhanced their ability to produce TGF-β1, thus validating the role of KRAS in the production of this cytokine. These data demonstrate an important role for the MEK–ERK–AP-1 signaling pathway in KRAS^Mut^-driven secretion of TGF-β1 in KRAS^Mut^ lung cancer. TGF-β1 secreted from KRAS^Mut^ cells increased Smad2/3 phosphorylation in an autocrine manner, which in turn activated the JNK1–SIRT1 pathway and consequently increased KRAS^Mut^ activity via enhanced KRAS^Mut^ deacetylation. These results indicate that KRAS^Mut^ facilitates the secretion of TGF-β1 by KRAS^Mut^ lung cancers, which is responsible for KRAS^Mut^ chemoresistance. By contrast, the inhibition of Smad2/3 in KRAS^Mut^ lung cancer resulted in a significantly reduced activity of KRAS^Mut^. Our findings suggest that Smad2/3 inhibition mitigates KRAS^Mut^ activity via a reduction in SIRT1 activity, thereby decreasing its ability to induce chemoresistance.

Natural products have historically been regarded as important resources of therapeutic agents. Over the past few decades, several natural compounds have been discovered that are now widely used as anticancer agents, including paclitaxel, vinblastine, camptothecin and oleuropein^57^. In this study, we did not focus on the anticancer activities of natural products by themselves because we have already reported that their combination with existing anticancer drugs as an adjuvant agent inhibits SIRT1, enhancing anticancer capacity^23^. Herein, we highlight the underlying mechanisms by which KWN-C downregulates SIRT1 activity and demonstrate the antitumor properties of CP and MTX when combined with KWN-C in various in vitro and in vivo models. Intriguingly, KWN-C suppressed both pSmad2/3 and pJNK levels, which subsequently downregulated the phosphorylation level of SIRT1^S27^ and SIRT1^S47^. Taken together, our findings demonstrate that oncogenic KRAS in lung cancer cells positively upregulates chemoresistance by inducing TGF-β1 secretion. We identified that TGF-β1–Smad2/3 increased KRAS^Mut^ activity via the JNK1–SIRT1 pathway, completely abrogating KWN-C. We believe that our findings have important implications for therapeutic interventions in patients with KRAS^Mut^ tumors.

Lung cancer represents an enormous health burden worldwide due to its high motility. However, many trials on KRAS^Mut^ lung cancer have either failed or not been successful, rendering *KRAS*^*Mut*^ NSCLC refractory to all targeted mono therapies so far. Consequently, there is a need to develop novel therapies to treat *KRAS*^*Mut*^ cancer. Our findings demonstrate that oncogenic KRAS in lung cancer cells positively upregulates chemoresistance by inducing TGF-β1 secretion via the MEK–ERK–AP-1 pathway. We also identified that TGF-β1 activates Smad2/3–JNK1 in an autocrine manner, which subsequently increases SIRT1 activity via pSIRT1^S27^ and SIRT1^S47^. Furthermore, an increase in SIRT1 activity was found to enhance KRAS^Mut^ activity via deacetylation. However, the KRAS^Mut^–TGF-β1–Smad2/3–JNK1–SIRT1 strong positive feedback loop may be a challenging therapeutic target. We confirmed that an SIRT1 activity inhibitor, KWN-C, could suppress the TGF-β1–Smad2/3 pathway with minimal toxicity. Thus, the combination therapy of SIRT1 activity inhibitor combined with existing anticancer drugs showed the greatest synergistic anticancer effects, rendering this approach a potential candidate as an adjuvant agent. These findings further support an effective combined regimen with a promising treatment strategy to sensitize the chemoresistance of KRAS^Mut^ NSCLC to clinical TGF-β–Smad2/3 inhibitors or SIRT1 activity inhibitors via concurrent chemotherapy with important implications for therapeutic interventions in patients with KRAS^Mut^ tumors (Fig. 8g).

## Supplementary information

Supplementary Information

## Data Availability

All data and materials are available upon request by contacting the corresponding author.
