# Supplementary material for: Targeting TGF-β–Smad2/3–JNK1-mediated SIRT1 activity overcomes the chemoresistance of KRAS mutation lung cancer
Source: Exp Mol Med. 2025 Sep 12;57(9):2022–39. doi: 10.1038/s12276-025-01536-8 (PMC12508081; doi:10.1038/s12276-025-01536-8)
Supplement: Supplementary file 1 — Supplementary Information [file 12276_2025_1536_MOESM1_ESM.pdf]

SUPPLEMENTARY FIGURE LEGENDS

Supplementary Fig. 1

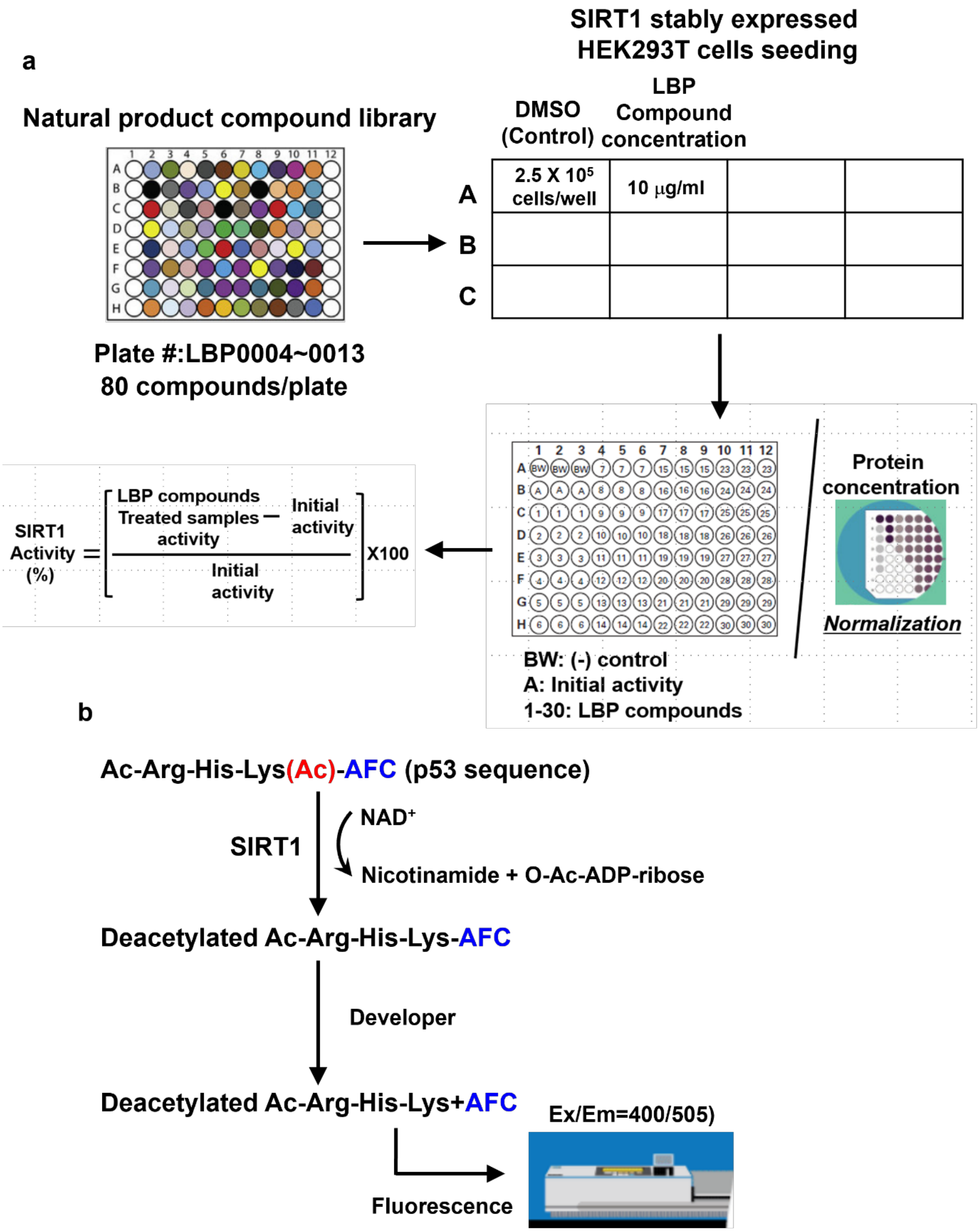

Supplementary Fig. 1 Schematic overview of SIRT1 activity measure with the natural product compound library.

a SIRT1 stably overexpressed HEK-293T cells were plated with a drug library consisting of natural product compounds. Cell lysates were measured by SIRT1 activity assay kit (Biovision, Ca# K324-100, Milpitas, CA, USA) for a SIRT1 activity dropout screen. **b** The acetylated p53-AFC substrate is deacetylated by SIRT1 in the presence of NAD^+ to generate the deacetylated p53-AFC substrate, nicotinamide and O-Acetyl-ADP Ribose. Cleavage of the deacetylated p53-AFC substrate by the developer releases the fluorescent group, which is detected fluorometrically at Ex/Em = 400/505 nm

Supplementary Fig. 2

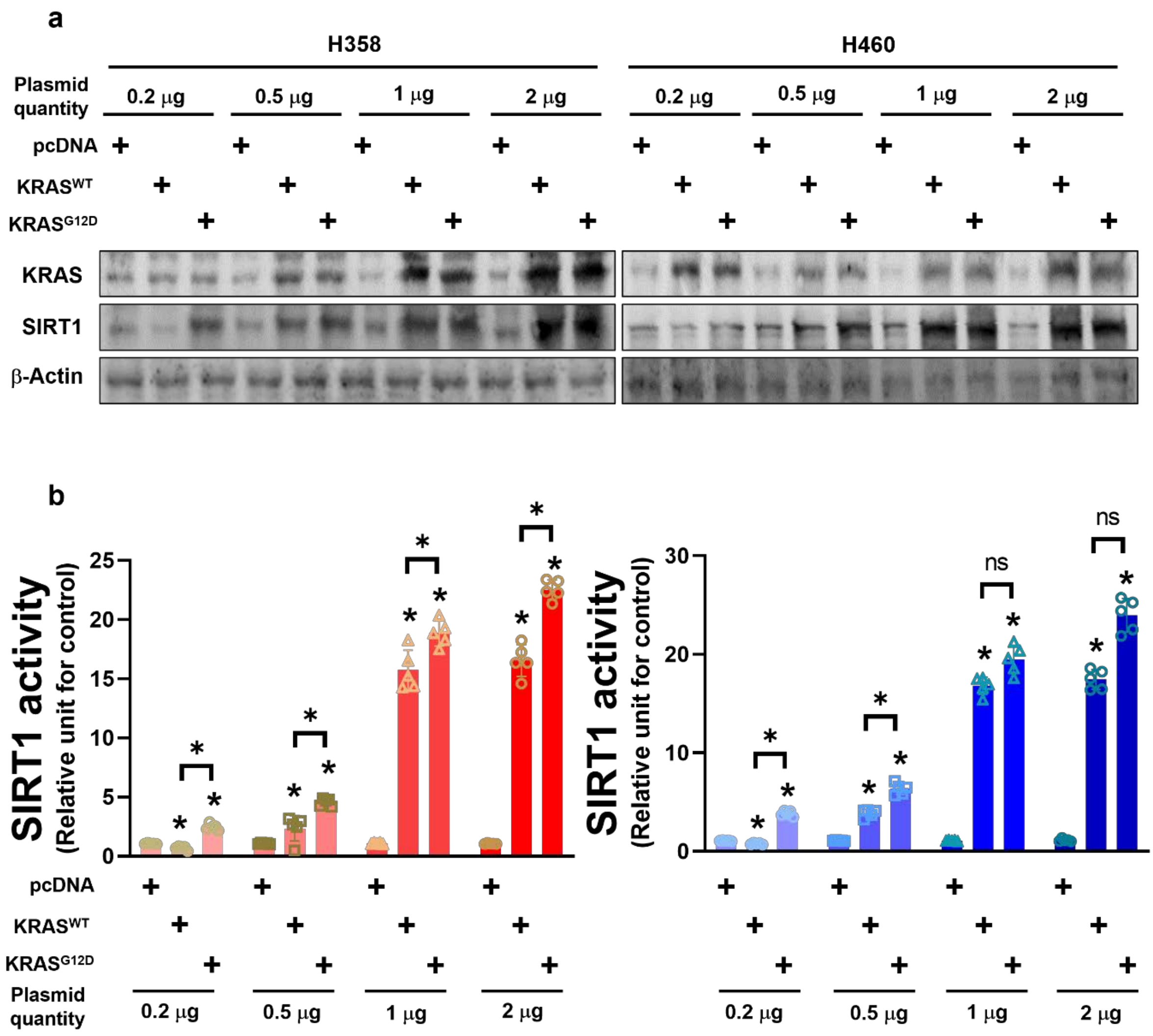

Supplementary Fig. 2 Dose-dependent transfection of KRAS^{WT} and KRAS^{Mut} reveals mutation-specific regulation of SIRT1 expression and activity.

a H358 and H460 cells were transfected with increasing amounts (0.2, 0.5, 1, and 2 μg) of pcDNA, KRAS^{WT}, or KRAS^{G12D} plasmids. KRAS and SIRT1 protein expression was measured by western blotting. β-actin was used as a loading control.

b SIRT1 enzymatic activity was assessed from the same transfected cell lysates. Student's *t*-test, mean ± SD; n = 5, *, $p < 0.05$.

Supplementary Fig. 3

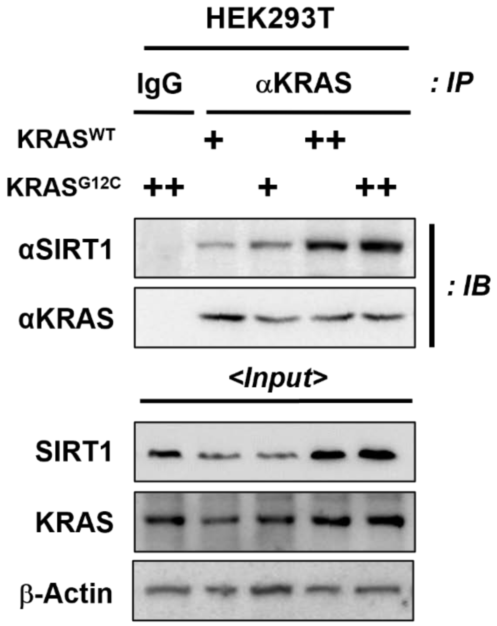

Supplementary Fig. 3 Mutant KRAS (G12C) exhibits stronger binding to SIRT1 than wild-type KRAS in HEK 293 cells.

HEK 293T cells were transfected with either KRAS^{WT} or KRAS^{G12C} 1 or 3 μg plasmids at equal DNA concentrations. Whole-cell lysates were collected 48 hours post-transfection and subjected to co-immunoprecipitation using an anti-KRAS antibody. Western blotting was performed with anti-SIRT1, anti-KRAS, and β-actin antibodies.

Supplementary Fig. 4

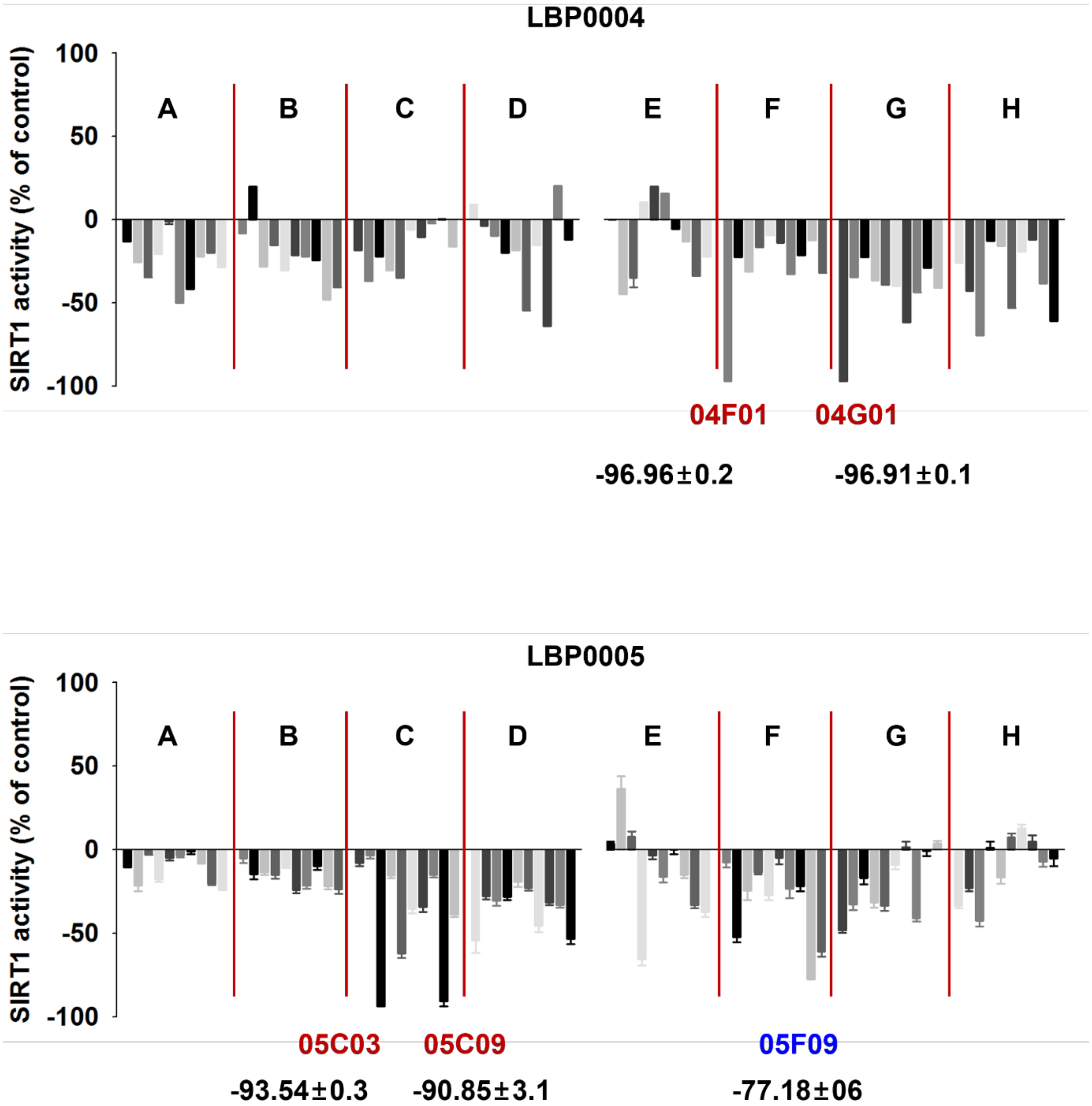

Supplementary Fig. 5

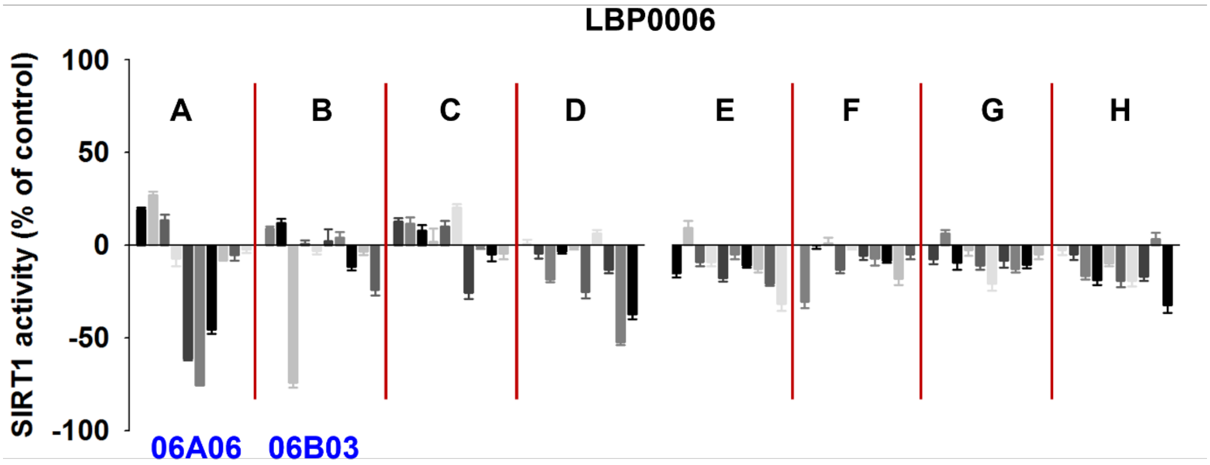

-75.28±0.7 -74.09±2.7

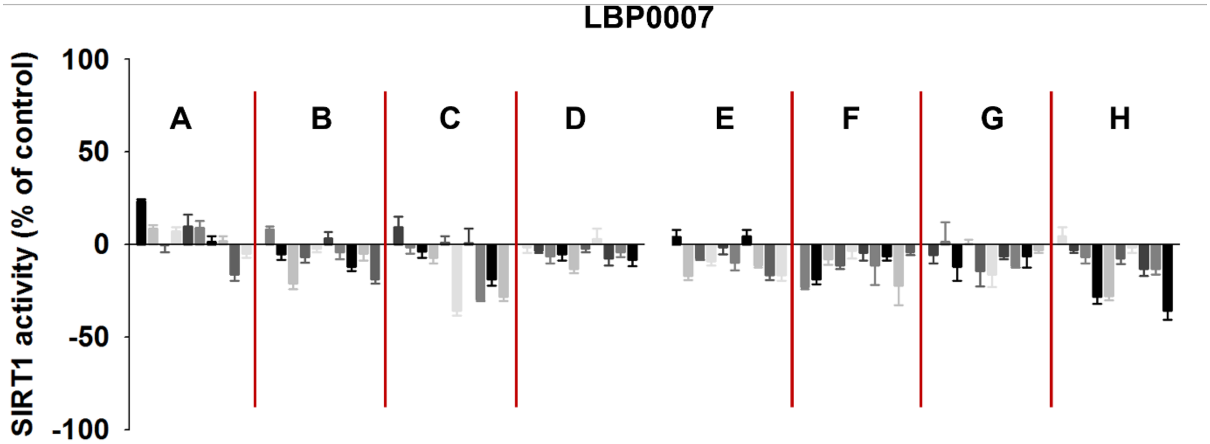

Supplementary Fig. 6

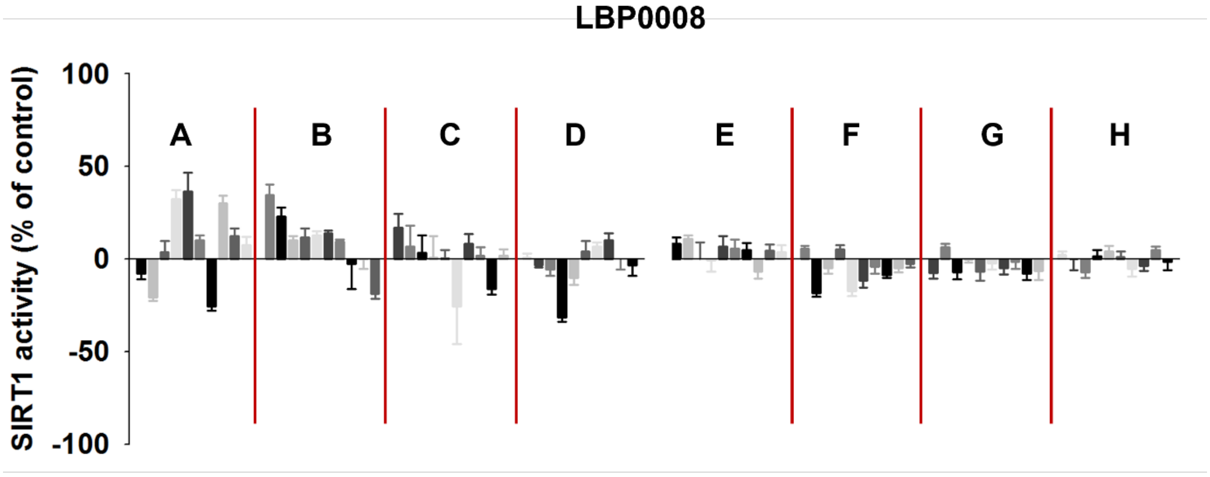

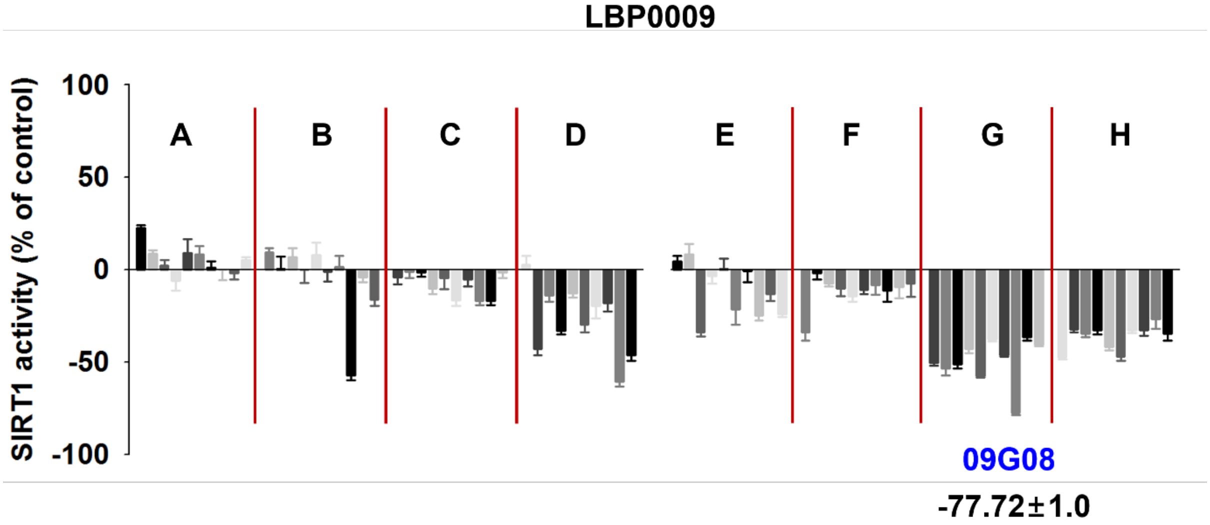

Supplementary Fig. 7

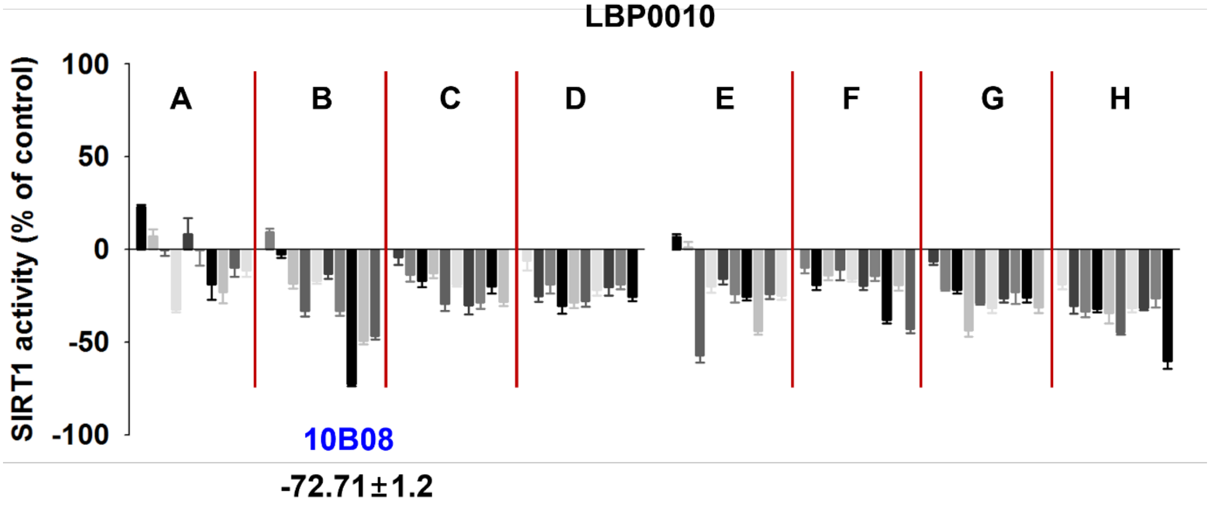

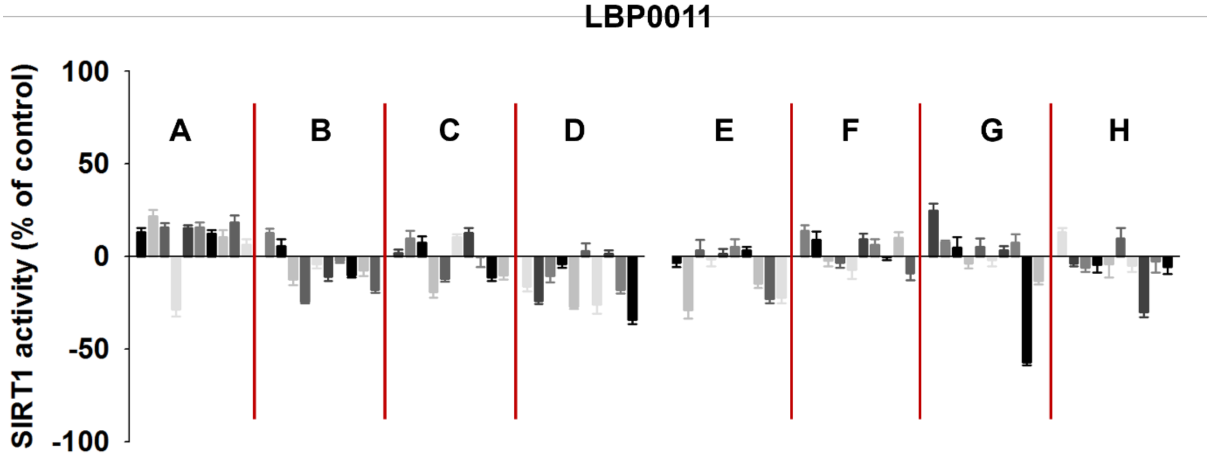

Supplementary Fig. 8

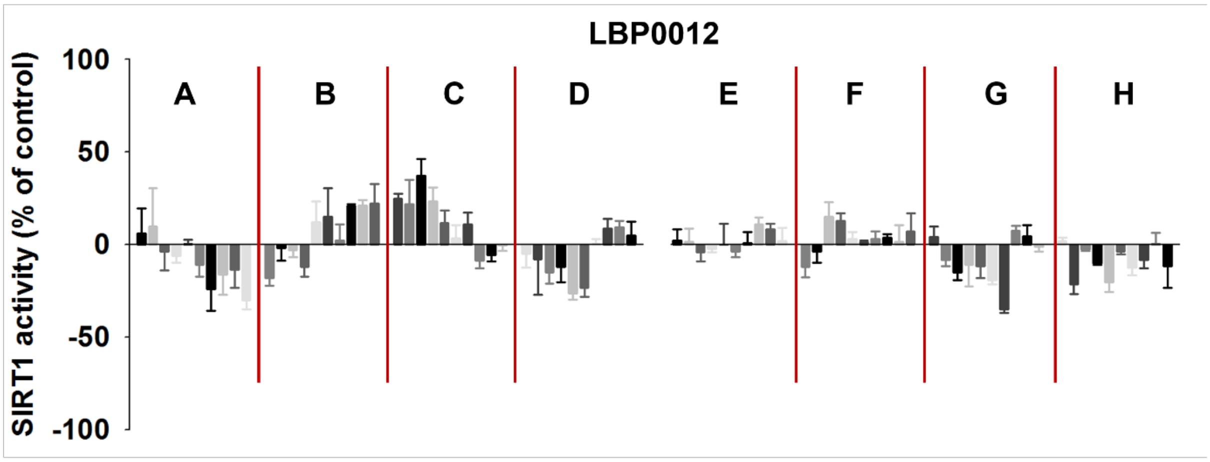

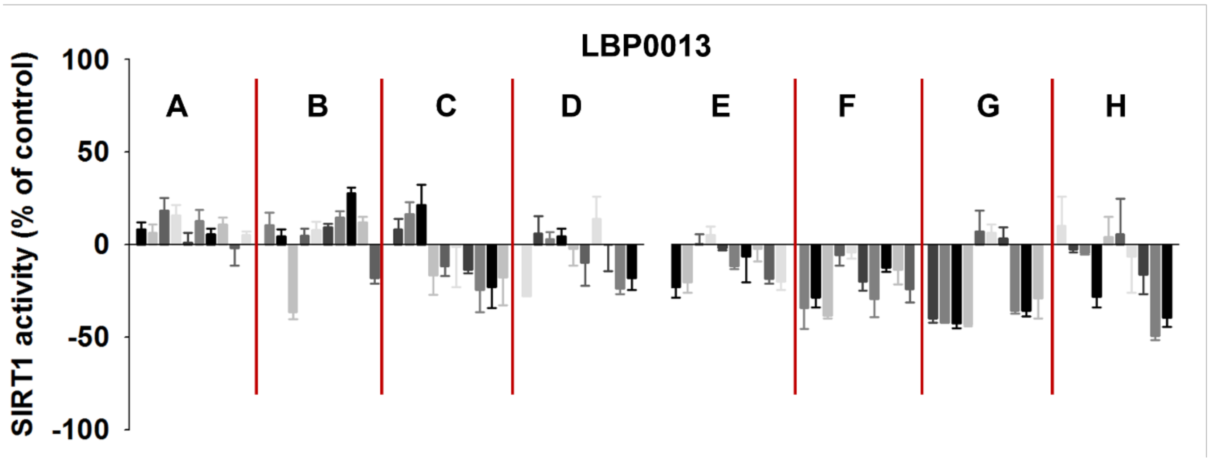

Supplementary Fig. 4, 5, 6, 7, and 8 Natural product compounds screening to identify compounds affecting SIRT1 activity

SIRT1 expressed HEK-293T cells were treated with 80 compounds per plate named from LBP0004 and LBP0005 (Supplementary Fig. 4), LBP0006 and LBP0007 (Supplementary Fig. 5), LBP0008 and LBP0009 (Supplementary Fig. 6), LBP0010 and LBP0011 (Supplementary Fig. 7), and LBP0012 and LBP0013 (Supplementary Fig. 8). SIRT1 activity were normalized by each well protein concentration. Student's *t*-test, mean ± SD; n = 6, *, $p < 0.05$.

Supplementary Fig. 9

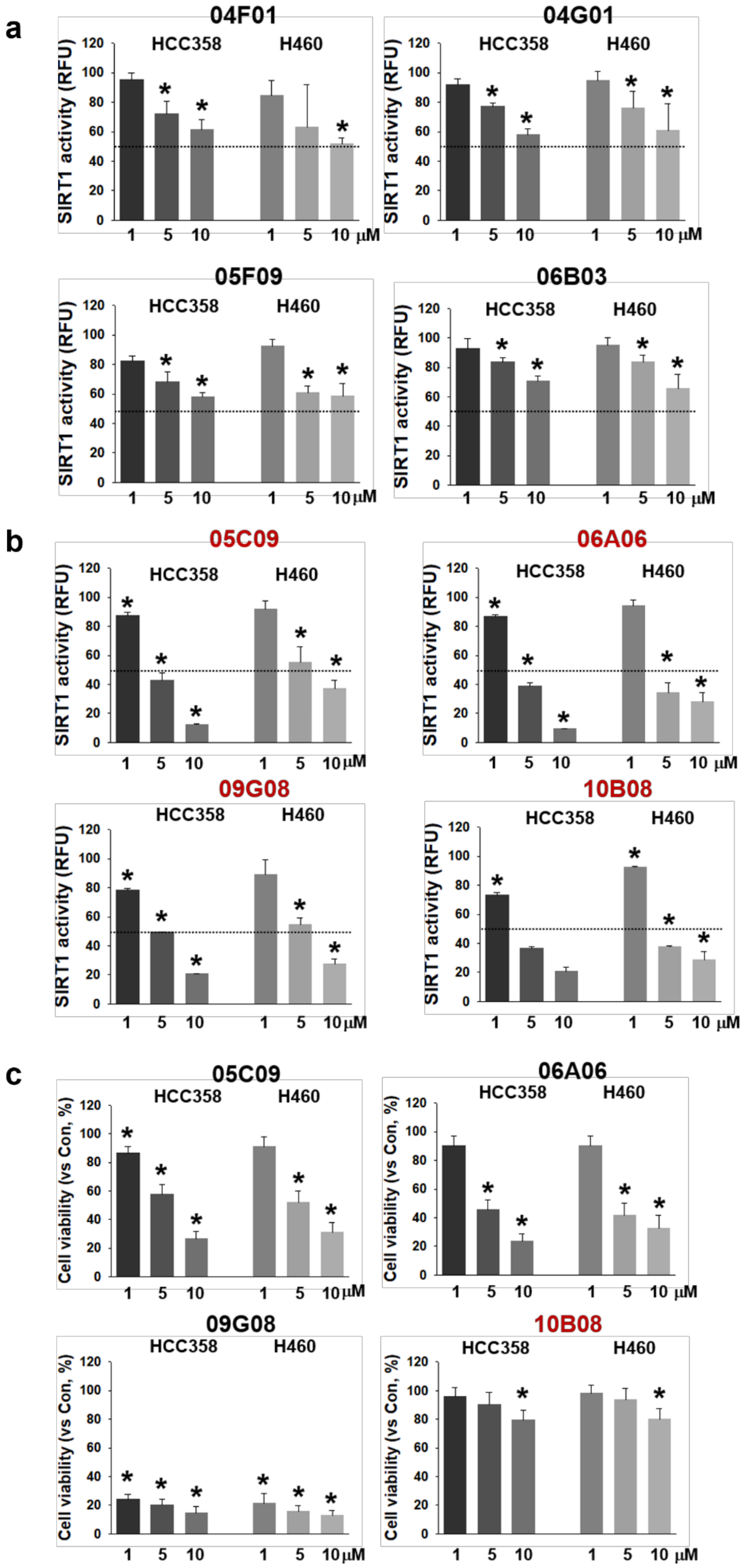

Supplementary Fig. 9 Check SIRT1 activity and cell viability with screened natural compounds reduced SIRT1 activity over 70%.

a and **b** H358 and H460 cells were treated with natural compounds does dependently (1, 5, and 10 μM). The cells were harvested with lysis buffer and SIRT1 activity assay was performed. Student's *t*-test, Mean ± SD; n = 6, *, $p < 0.05$. **c** H358 and H460 cells were treated with same dose in supplementary Fig. 9a. Cell proliferation was assessed using the MTS assay three days after the drug treatment. Student's *t*-test, Mean ± SD; n = 6, *, $p < 0.05$.

Supplementary Fig. 10

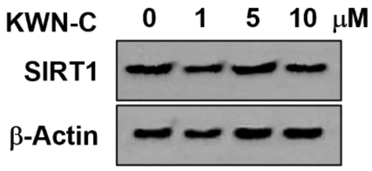

Supplementary Fig. 10 SIRT1 protein levels measurement

H358 cells were treated with KWN-C dose dependently and then cell lysates were immunoblotted with anti-SIRT1 antibody.

Supplementary Fig. 11

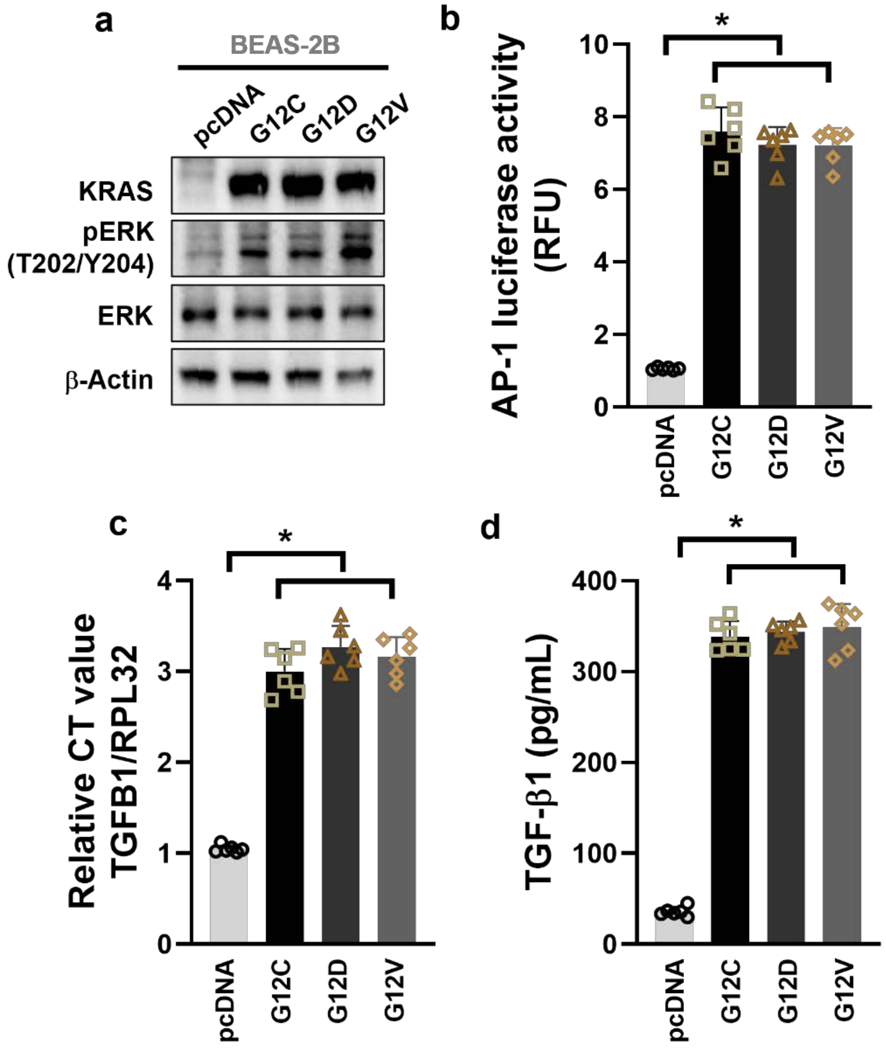

Supplementary Fig. 11 Enhanced TGF-β1 in KRAS^{Mut} cells dependents on MEK-ERK-AP1 pathway.

a Normal human bronchial epithelial cell (BEAS-2B) cells were transfected with pcDNA, KRAS G12C, G12D, and G12V plasmids (2 μg) and then harvested with lysis buffer, and subjected to western blotting with KRAS, anti-pERK, ERK, and β-actin antibodies. **b** A luciferase assay was performed to measure the AP-1-mediated transcriptional regulatory activity with cell lysates transfected with pcDNA, KRAS G12C, G12D, G12V, and AP-1 luciferase plasmids (2 μg). Student's *t*-test, mean ± SD; n = 6, *, $p < 0.05$. **c** TGFB1 mRNA expression was measured by quantitative real-time PCR with same cell lines and transfection conditions of supplementary Fig. 11a. *RPL32* was used as internal control and for normalization. Student's *t*-test, mean ± SD; n = 6, *, $p < 0.05$. **d** The medium of BEAS-2B cells with transfected pcDNA and KRAS^{Mut} plasmids were changed by FBS-free medium before cell harvest 24 h. Conditioned medium was collected and concentrated by amicon ultra-15 tube, and the total TGF-β1 levels were measured using enzyme-linked immunosorbent assay.

Supplementary Fig. 12

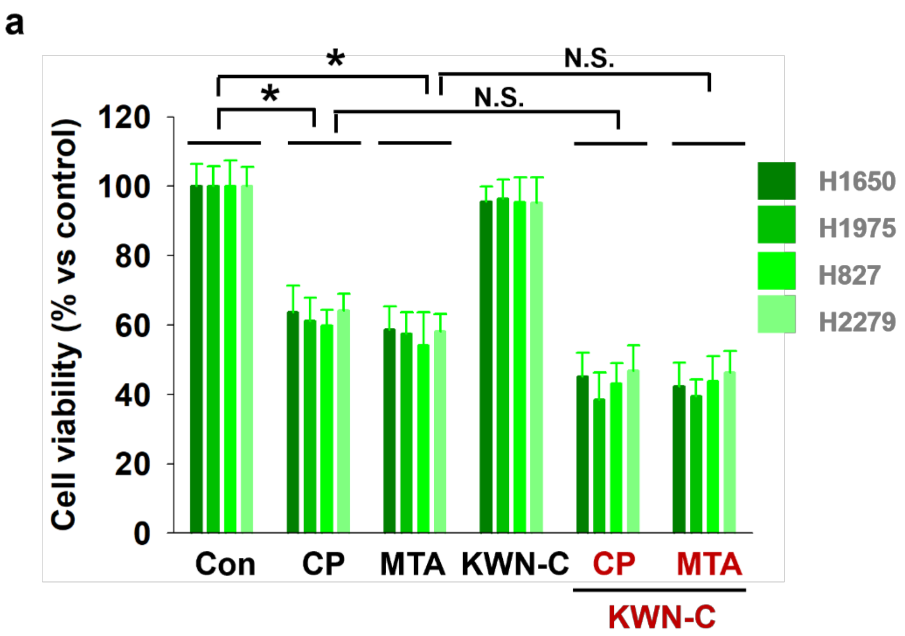

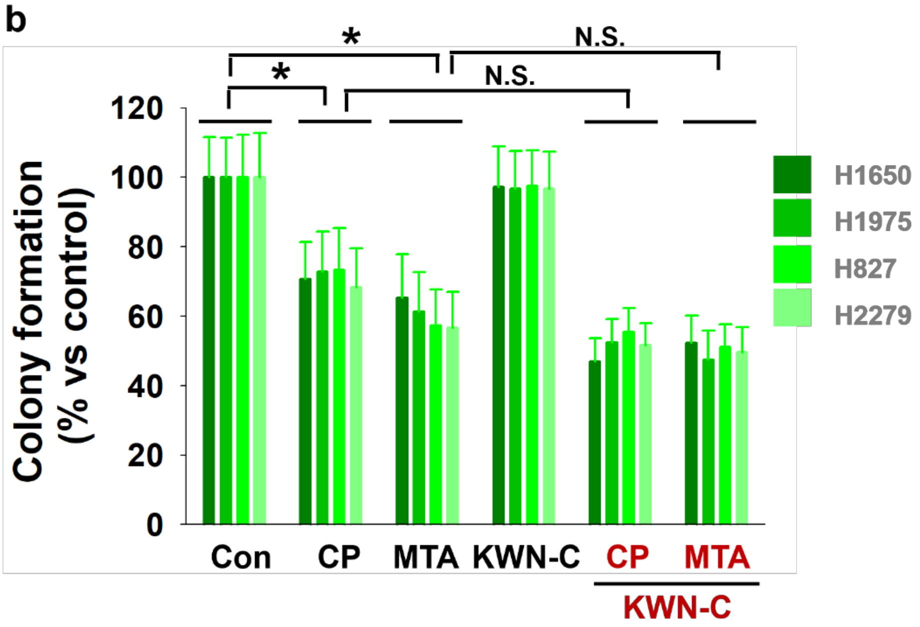

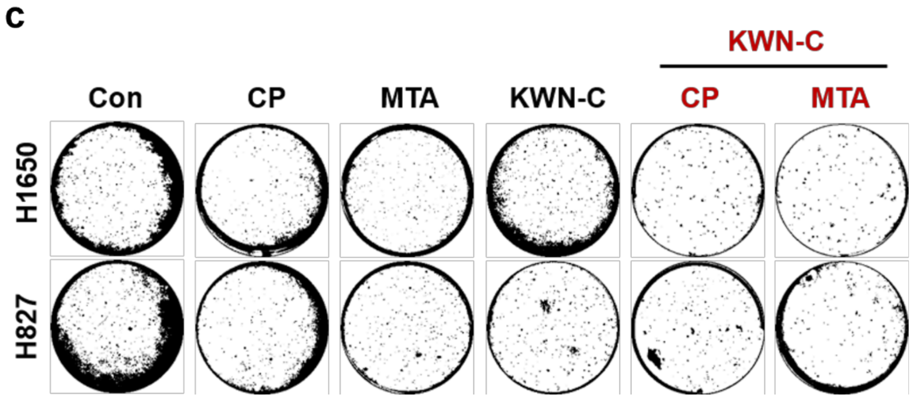

Supplementary Fig. 12 $EGFR^{Mut}$ cells measured cell viability and colony formation after treatment with CP, MTA, and/or KWN-C.

a H1650, H1975, H827, and H2279 cells were treated with CP (5 μM), MTA (5 μM), and/or KWN-C (10 μM), and cell proliferation was measured by the MTS assay three days after drug treatment. Student's *t*-test, mean ± SD; n = 6, *, $p < 0.05$. **b** $EGFR^{Mut}$ ells were seeded with 0.5% top agar and cultured in a mixture of fresh medium with drugs as described in supplementary Fig. 12a. Student's *t*-test, mean ± SD; n = 6, *, $p < 0.05$. **c** Cell colonies were stained with crystal violet and counted per 3.8 cm^2. Representative colony images were showed.

Supplementary Fig. 13

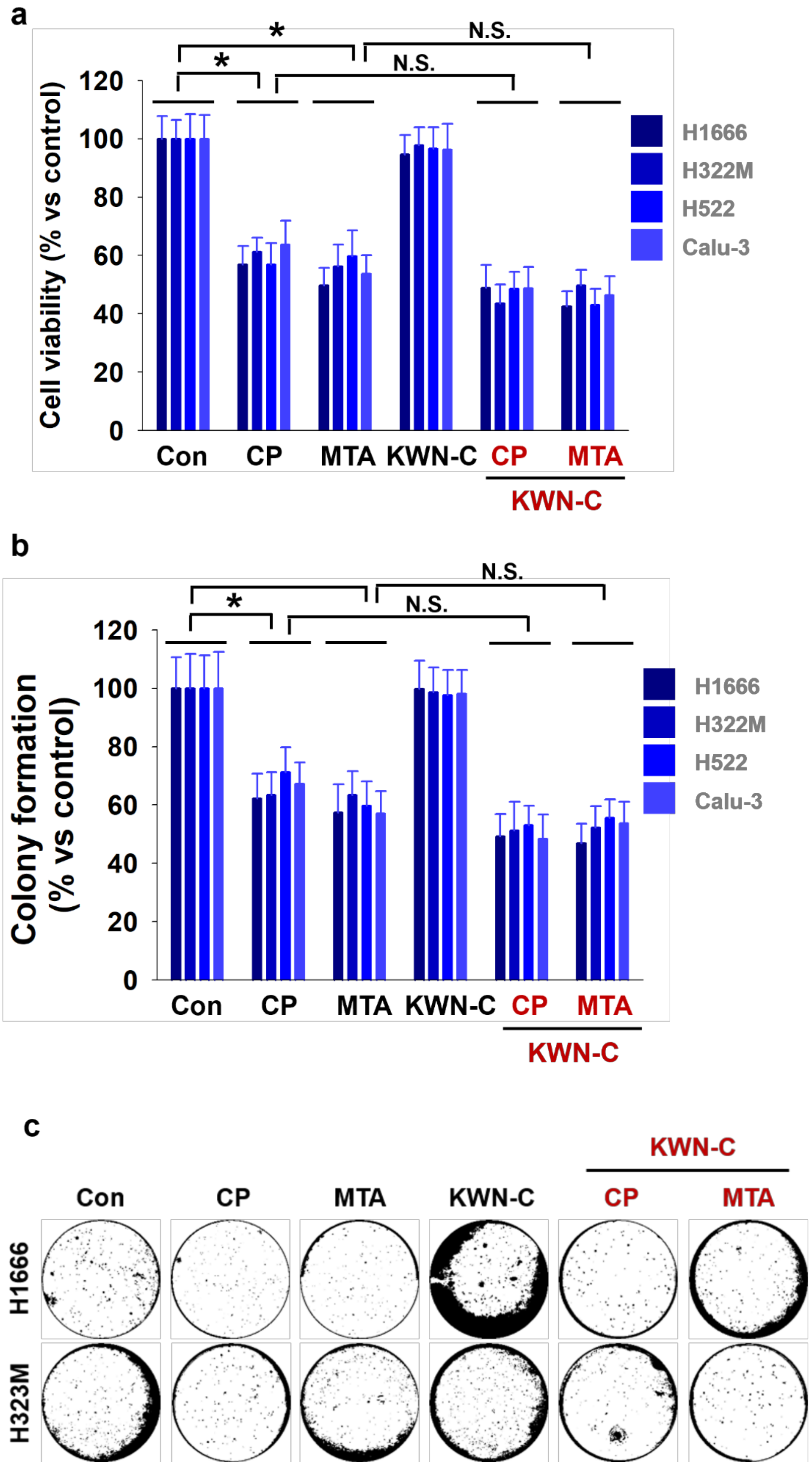

Supplementary Fig. 13 $KRAS^{WT}$ and $EGFR^{WT}$ cells measured cell viability and colony formation after treatment with CP, MTA, and/or KWN-C.

a H1666, H322M, H522, and Calu-3 cells were treated with CP (5 μM), MTA (5 μM), and/or KWN-C (10 μM), and cell proliferation was measured by the MTS assay three days after drug treatment. Student's *t*-test, mean ± SD; n = 6, *, $p < 0.05$. **b** $KRAS^{WT}$ and $EGFR^{WT}$ cells were seeded with 0.5% top agar and cultured in a mixture of fresh medium with drugs as described in supplementary Fig.13a. Student's *t*-test, mean ± SD; n = 6, *, $p < 0.05$. **c** cell colonies were stained with crystal violet and counted per 3.8 cm^2. Representative colony images were showed.

Supplementary Fig. 14

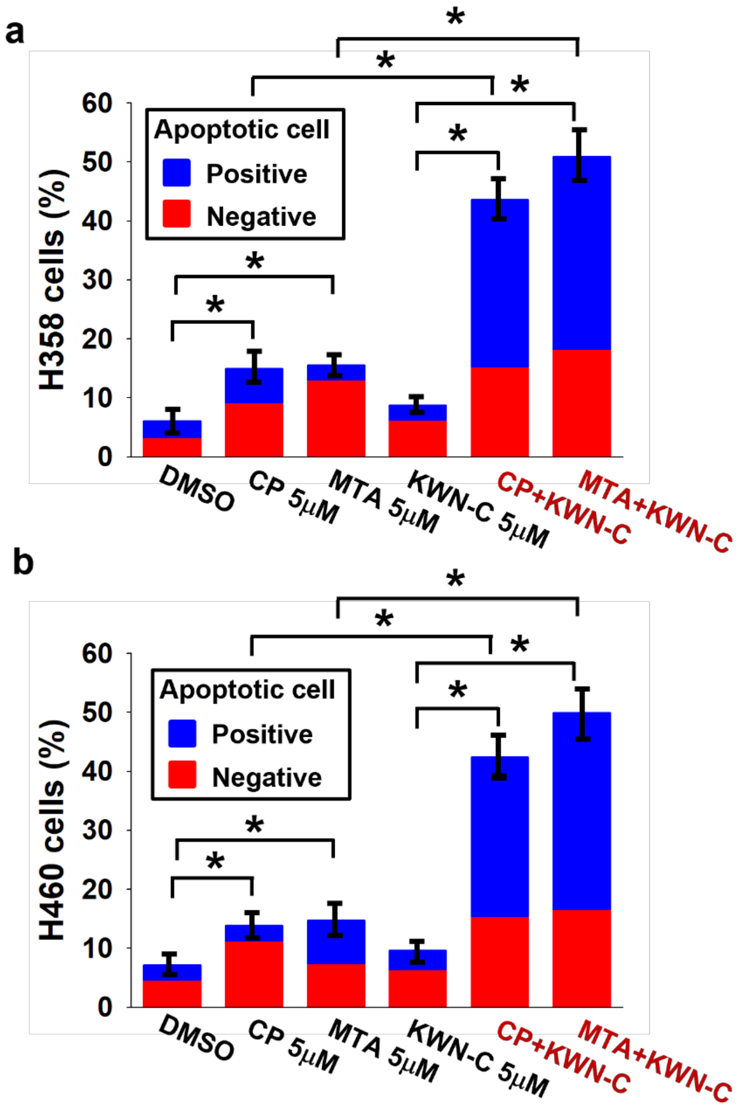

Supplementary Fig. 14 KRAS[Mut] cells measured cell apoptosis after treatment with CP, MTA, and/or KWN-C.

a and **b** The percentage of apoptotic H358 and H460cells (blue column) and non-apoptotic cells (red column) in Fig. 5d between CP, MTA, and/or KWN-C were statistically compared. Student's *t-test*, mean ± SD; n = 6, * $p < 0.05$.

Supplementary Fig. 15

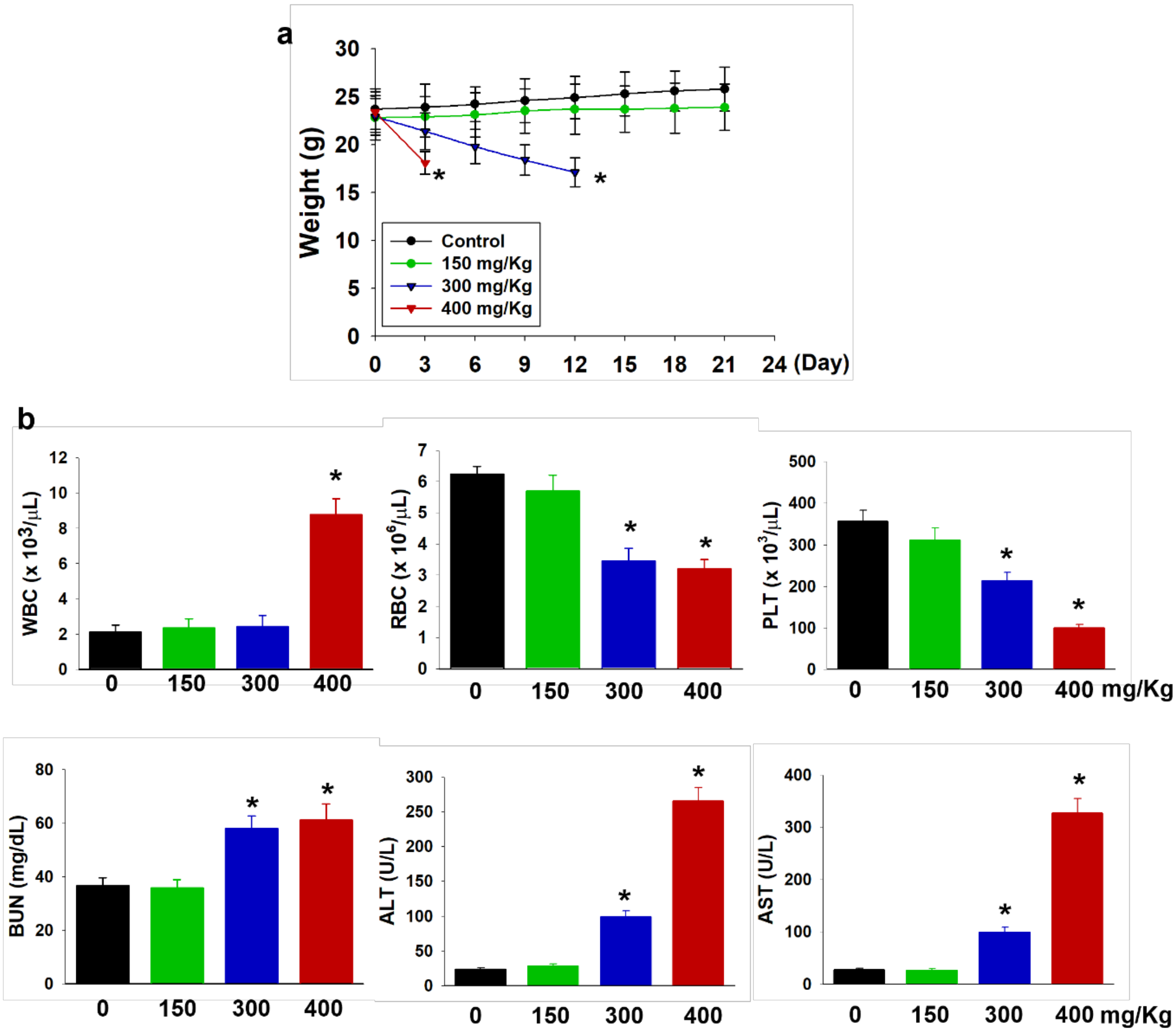

Supplementary Fig. 15 Determination of single dose maximum tolerated dose (MTD)

a Nu/Nu nude mice were treated with single dose (i.e. 0, 150 mg/Kg, 300 mg/Kg, and 400 mg/Kg) KWN-C via i.p. After treatment, the body weight of mice was measured every 3 days for 3 weeks. Student's *t*-test, mean ± SEM; n = 6, *, $p < 0.05$. **b** Blood analysis of mice after treatment with single dose(s) of KWN-C. Student's *t*-test, mean ± SEM; n = 6, *, $p < 0.05$.

SUPPLEMENT TABLE LEGENDS

Supplementary Table. 1

Library #	Systemic Name	Formula	SIRT1 activity
04F01	Dioscin	$C_{45}H_{72}O_{16}$ (M.W 868)	-96.96±0.2
04G01	Xanthoangelol	$C_{31}H_{20}O_{10}$ (M.W 552)	-96.91±0.1
05C03	ent-7β-hydroxy-15-oxokaur-16-en-18-yl acetate		-93.54±0.3
05C09	Mulberrofuran C	$C_{34}H_{28}O_{9}$ (M.W 580)	-90.85±3.1
05F09	(E)-5-Hydroxy-3-7-methoxy-(2'-hydroxybenzylidene)-4-chromanone	$C_{17}H_{14}O_{5}$ (M.W 331)	-77.18±06
06A06	Kurarinone	$C_{26}H_{36}O_{6}$ (M.W 868)	-75.28±0.7
06B03	Sophoraflavanone G (vexibinol)		-74.09±2.7
09G08	Frugoside		-77.72±1.0
10B08	Kuwanon C	$C_{25}H_{26}O_{6}$ (M.W 422)	-72.71±1.2

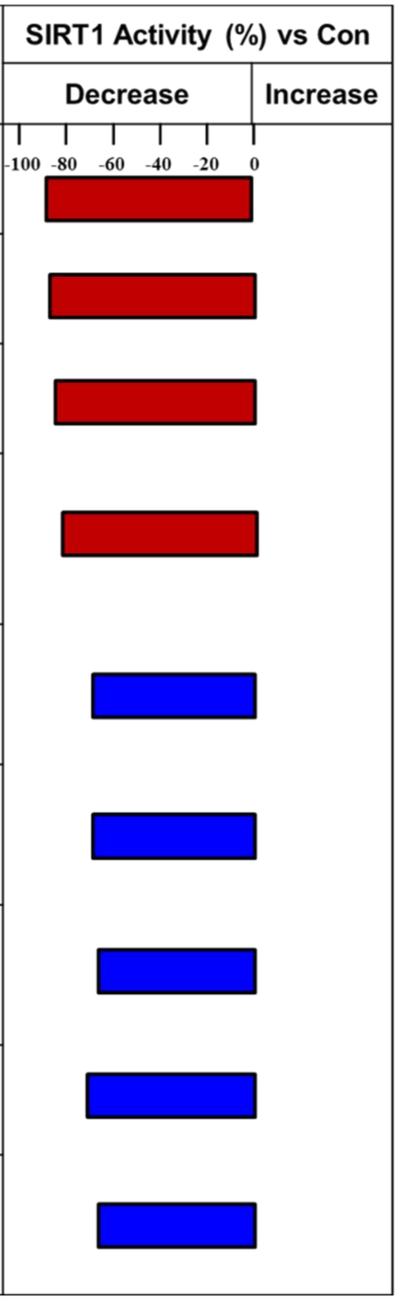

Supplementary Table. 1 Drugs summary of inhibiting SIRT1 activity over 70%

Nine natural compounds were screened with inhibiting SRIT1 activity. Top four red natural compounds inhibited SIRT1 activity over 80% and next five blue natural compounds inhibited SIRT1 activity between 80% and 70%.
